# Supermarket policies on less-healthy food at checkouts: Natural experimental evaluation using interrupted time series analyses of purchases

**DOI:** 10.1371/journal.pmed.1002712

**Published:** 2018-12-18

**Authors:** Katrine T. Ejlerskov, Stephen J. Sharp, Martine Stead, Ashley J. Adamson, Martin White, Jean Adams

**Affiliations:** 1 Centre for Diet and Activity Research, MRC Epidemiology Unit, University of Cambridge, Cambridge, United Kingdom; 2 Institute for Social Marketing, Faculty of Health Sciences and Sport, University of Stirling, Stirling, United Kingdom; 3 Institute of Health & Society and the Human Nutrition Research Centre, Newcastle University, Newcastle upon Tyne, United Kingdom; Carolina Population Center, UNITED STATES

## Abstract

**Background:**

In response to public concerns and campaigns, some United Kingdom supermarkets have implemented policies to reduce less-healthy food at checkouts. We explored the effects of these policies on purchases of less-healthy foods commonly displayed at checkouts.

**Methods and findings:**

We used a natural experimental design and two data sources providing complementary and unique information. We analysed data on purchases of small packages of common, less-healthy, checkout foods (sugary confectionary, chocolate, and potato crisps) from 2013 to 2017 from nine UK supermarkets (Aldi, Asda, Co-op, Lidl, M&S, Morrisons, Sainsbury’s, Tesco, and Waitrose). Six supermarkets implemented a checkout food policy between 2013 and 2017 and were considered intervention stores; the remainder were comparators.

Firstly, we studied the longitudinal association between implementation of checkout policies and purchases taken home. We used data from a large (*n* ≈ 30,000) household purchase panel of food brought home to conduct controlled interrupted time series analyses of purchases of less-healthy common checkout foods from 12 months before to 12 months after implementation. We conducted separate analyses for each intervention supermarket, using others as comparators. We synthesised results across supermarkets using random effects meta-analyses. Implementation of a checkout food policy was associated with an immediate reduction in four-weekly purchases of common checkout foods of 157,000 (72,700–242,800) packages per percentage market share—equivalent to a 17.3% reduction. This decrease was sustained at 1 year with 185,100 (121,700–248,500) fewer packages purchased per 4 weeks per percentage market share—equivalent to a 15.5% reduction. The immediate, but not sustained, effect was robust to sensitivity analysis.

Secondly, we studied the cross-sectional association between checkout food policies and purchases eaten without being taken home. We used data from a smaller (*n* ≈ 7,500) individual purchase panel of food bought and eaten ‘on the go’. We conducted cross-sectional analyses comparing purchases of common checkout foods in 2016–2017 from supermarkets with and without checkout food policies. There were 76.4% (95% confidence interval 48.6%–89.1%) fewer annual purchases of less-healthy common checkout foods from supermarkets with versus without checkout food policies.

The main limitations of the study are that we do not know where in the store purchases were selected and cannot determine the effect of changes in purchases on consumption. Other interventions may also have been responsible for the results seen.

**Conclusions:**

There is a potential impact of checkout food polices on purchases. Voluntary supermarket-led activities may have public health benefits.

## Abstract 2

Jean Adams and colleagues describe the effects of UK supermarket healthy eating food policies on food purchases both for home and on the go.

## Author summary

**Why was this study done?:**

- Supermarkets often make changes in-store that can influence what people buy. Some of these changes may lead to improvements in the quality of people’s diets.
- A number of UK supermarkets have introduced policies on what food should and should not be displayed at their checkouts.
- Previous work has found that supermarkets with checkout food policies have less food at their checkouts, and the food that is there is likely to be healthier than the food in supermarkets without checkout food policies. It is not known whether these changes lead to changes in what people buy.

**What did the researchers do and find?:**

- We used data from more than 30,000 UK households who recorded all the food they bought and brought home during 2013–2017. We found that about 17% fewer small packages of sugary confectionary, chocolate, and potato crisps were bought and taken home from supermarkets immediately after they announced a checkout food policy. One year after, the difference was about 16% fewer.
- We also used data from about 7,500 people who recorded all the food they bought and ate without bringing home. In 2016 and 2017, about 76% fewer small packages of sugary confectionary, chocolate, and potato crisps were bought and eaten ‘on the go’ from supermarkets with checkout food policies compared to those without.

**What do these findings mean?:**

- Because this is not a trial, we cannot be sure that the changes in purchases we recorded are due to checkout food policies. Nor do we know if there were any other changes in what people bought or ate. However, these policies could help people to eat better.

## Introduction

In most industrialised countries, large supermarket chains have captured the majority of the grocery market [1]. Although retailers are clearly responsive to consumer demand, they also play a major role in shaping food preferences and purchasing behaviour [1–3].

Retail practices such as product displays, placement, promotions, and pricing are an important component of the context for consumers’ choices in stores [4], and there is evidence that these can encourage purchases [1,3,5]. One example of in-store marketing is the positioning of food at supermarket checkouts. Checkouts provide a unique location for prompting purchases, as all customers have to pass through them to pay and may spend considerable time in queues. Internationally, the majority of food at supermarket checkouts is of the type that governmental recommendations do not encourage greater consumption of [6–10].

In response to consumer demand and calls by governments and civil society to play a more active public health role, supermarket-led activities with the potential to promote healthier diets are becoming more common [1]. In the UK, advocacy groups [11–13], the media [14], and researchers [10] have voiced ongoing concern about the nutritional quality of food at supermarket checkouts. Over the last decade, many UK supermarket groups have pledged to provide healthier checkout foods [13,15]. These are voluntary, supermarket-led commitments unrelated to specific government action. Although these policies are rarely identified by supermarkets as having an explicit public health intent, they have the potential to impact public health via impacts on purchasing and consumption of less-healthy foods.

We previously demonstrated that supermarkets with checkout food policies displayed fewer checkout foods, with a lower proportion of these foods being less-healthy (as defined by the UK Food Standards Agency’s Nutrient Profile Model) [16], compared with supermarkets with no policies [15,17]. Furthermore, in one city, we found no evidence that foods near, but not at, checkouts in supermarkets with checkout food policies were more likely to be less-healthy than those in supermarkets without checkout food policies [17]. This suggests that supermarkets do not necessarily undermine their policies by moving less-healthy [16] checkout food to the immediate vicinity of checkouts. These findings suggest that voluntary actions by supermarkets can be in line with public health goals. However, the impact of these policies on purchasing behaviour has not been determined. It is, for example, possible that checkout food policies simply displace purchasing of products from checkout areas to elsewhere in stores.

In this paper, we explore the immediate, sustained, and longer-term associations between the introduction of supermarket checkout food policies and purchases of foods commonly displayed at checkouts.

## Methods

### Outline of evaluative strategy

A well-conducted randomised controlled trial would be the strongest test of the effect of supermarket checkout food policies. However, these policies were not researcher-led and were introduced rapidly without the opportunity for randomisation, and formal control groups may have been unacceptable to retailers. Instead, we used a pragmatic, natural experimental design [18] using data that were collected for other purposes. We conducted two complementary sets of analyses using different data sources to address our aims. Alone, neither data source is ideal. Together, they provide two complementary and unique datasets.

Firstly, we used data on food purchases brought into the home reported by a large UK commercial household purchase panel (data available since 2013). We performed controlled interrupted time series analyses exploring four-weekly purchases of common checkout foods (defined below) before and after implementation of checkout food policies. We conducted separate analyses for each supermarket group that implemented a checkout food policy and synthesised results at 4 weeks and 12 months post implementation using meta-analysis. We refer to these as the ‘longitudinal’ analyses, capturing immediate and sustained effects. Although these data capture a large proportion of purchases, they do not capture purchases of items that are consumed before being brought home.

To better capture purchases not brought home, we additionally used data on food purchases bought and eaten ‘on the go’ without ever being brought home, using a smaller UK commercial individual purchase panel. However, as these data were only available in 2016–2017, longitudinal analyses were not possible. Instead, we conducted cross-sectional analyses of purchases of common checkout foods in 2016–2017 comparing supermarkets with and without checkout food policies at that time. We refer to this as the ‘cross-sectional’ analysis, capturing long-term effects.

### Supermarkets and checkout food policies

Nine national supermarket groups (referred to in this paper as supermarkets) covering all 14 store formats associated with these supermarkets and representing more than 90% of the UK grocery market share [19,20] were included: Aldi, Asda, Coop, Lidl, M&S, Morrisons, Sainsbury’s, Tesco, and Waitrose. As our intention was to study associations between checkout food policies and purchases rather than ‘name and shame’ particular supermarkets, supermarkets are anonymised throughout.

Intervention supermarkets were defined as those that changed their checkout food policy between January 2014 (1 year after purchasing data became available) and July 2016 (1 year before we obtained data). Of the nine supermarkets considered, six met the criteria for being intervention supermarkets. A further two implemented checkout food policies before the study period began, and another had no checkout food policy throughout the study period [20]; together, these three were used as comparators that did not change their checkout food policies during the study period.

Information on checkout food policies was from our previous survey [15]. We first searched supermarkets’ annual reports, web pages, and press releases for relevant information. If this did not provide the detail we needed, we contacted supermarket customer services by letter, phone, or email. As a last resort, we used information in newspapers or other secondary sources. Checkout food policies were categorised into three groups as described previously [15]: ‘clear and consistent’ policies, ‘vague or inconsistent’ policies, and no policy. Clear and consistent policies were those that provided clear and specific information on which products would be removed and what products they should be replaced with (e.g., sweets and chocolate replaced with ‘healthier options including dried fruit, nuts, juices and water’) and which applied to all checkouts within a supermarket group or format. Vague or inconsistent policies were those that provided vague and nonspecific information on products to be removed or introduced ‘and/or’ policies that did not apply to all checkouts within a group or format (e.g., ‘limit display of confectionary to one in three checkouts’, no information on replacement products provided). Policies were heterogeneous in terms of the specifics of foods to be removed, replacement foods, and checkouts they applied to. Of the six intervention supermarkets, three introduced clear and consistent policies, and three introduced vague or inconsistent policies.

One comparator supermarket introduced a vague or inconsistent policy in their large-format stores in 2004 but not in their convenience stores. In the longitudinal analyses, these two store formats were combined because the small volume of four-weekly purchases in the convenience stores was too variable to provide reliable data, and the supermarket was used as a comparator. In the cross-sectional analysis using annual data, the two store formats were included separately.

### Definition of common checkout foods

Common checkout foods were selected based on our large survey of checkout food in 69 branches of included supermarkets [15]. Here we focus on the most common food categories from that survey that featured the highest proportion of less-healthy foods (as defined by the Food Standards Agency’s Nutrient Profile Model) [16]: sugary confectionery (i.e., sweets or candy; present in 31% of customer checkout journeys, with 97% being less-healthy), chocolate (present in 23% of checkout journeys, with 100% less-healthy), and potato crisps (i.e., potato chips; present in 21% of checkout journeys, with 71% less-healthy). As neither purchase panel identified where in the store products were selected, we focused on single-serve and smaller package sizes, which are more likely to be found at checkouts [15]. Thus, in both analyses, we included purchases of sugary confectionery in single-unit packages of ≤225 g, chocolate in packages of ≤125 g, and crisps in packages of ≤50 g. Purchases in all three groups were aggregated for analyses and are collectively termed ‘common checkout foods’ throughout.

### Purchase data, market share, and demographic characteristics of customers

For the longitudinal analyses, supermarket-specific data on purchases of common checkout foods were extracted from Kantar Worldpanel’s ‘Take-home’ panel (https://www.kantarworldpanel.com/global/Consumer-Panels/FMCG). This is a commercial, continuously refreshed panel of UK households (*n* ≈ 30,000). Participating households record all food and beverage purchases brought into the home, using an electronic scanner. Information captured includes purchase location, product line, and package size. Using quota sampling, the panel is broadly representative of the UK in terms of region, occupational social class, age of main shopper, and number of children in the household. Households receive monetary incentives for taking part, and quality control procedures exclude those that do not record minimum purchase volume and spend criteria. Most households stay in the panel for 2–3 years. Data from this panel have been found to reflect data from the Living Costs and Foods Survey—a government-funded cross-sectional household consumption survey [21].

**Fig 1:**
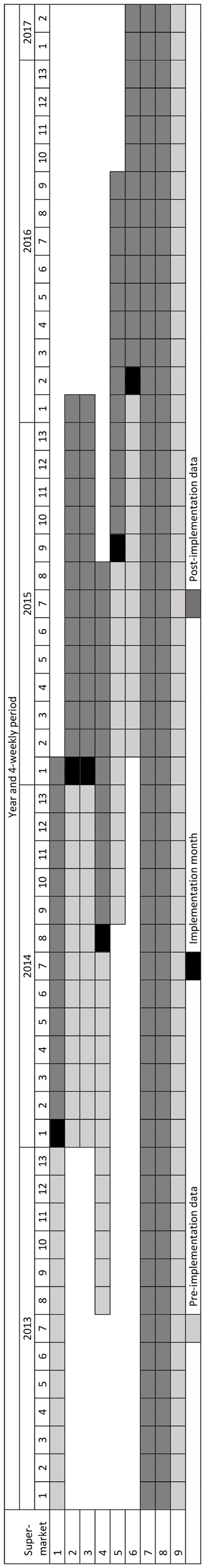
Temporal availability of data used in longitudinal analysis, by supermarket.

We obtained data on the number of packages of common checkout foods purchased from each included supermarket, aggregated into four-weekly periods and weighted and uplifted by Kantar to represent the total UK market (*n* = 27,385,050 households). We did not have access to household-level purchase data. For intervention supermarkets, we obtained data for the 13 four-weekly periods before and 13 four-weekly periods after implementation (26 data points). For comparator supermarkets, we obtained data for the full period January 2013–February 2017 (54 data points) (see Fig 1).

For the cross-sectional analysis, supermarket-specific data on purchases of common checkout foods were extracted from Kantar Worldpanel’s ‘Out of Home’ panel (https://www.kantarworldpanel.com/global/Consumer-Panels/Out-of-Home). This is a subsample of individuals in households in the ‘Take-home’ panel (*n* ≈ 7,500 individuals). These individuals additionally record all purchases that are consumed without being brought home using a mobile phone application. The ‘Out of Home’ panel was initiated in September 2015. We obtained data on the number of packages of common checkout foods purchased, aggregated into annual periods for 2016 and 2017 and weighted and uplifted by Kantar to represent the total UK market that the panel represents (*n* = 50,398,000 individuals aged 13–79 years). We did not have access to individual-level data or purchases over a smaller unit of time than 1 year.

Different supermarkets have different market shares. Thus, similar absolute changes in number of packages purchased from different supermarkets may represent different relative changes. To take account of this, we obtained rolling 12-weekly market share for each supermarket, also from Kantar. No information on market share was provided for one intervention supermarket, so we estimated it from 2017 annual reports.

Supermarkets also differ in terms of the demographic composition of their customers. To adjust for this in the cross-sectional analysis, we obtained information on the percentage of grocery spend by shoppers in different social grades and age groups in each supermarket from Kantar. Occupational social grade of the highest household earner was based on the Market Research Society coding [22] (AB [most affluent], C1, C2, D, and E [least affluent, unemployed, and retired]). Age was of the main household shopper (<28 years, 28–34 years, 35–44 years, 45–54 years, 55–64 years, and 65+ years). A ‘shopper mean social grade’ variable for each supermarket was calculated as the weighted mean value of social grade (social grade AB assigned a value of 5, C1 assigned a value of 4, and so on), using the proportion of grocery spend by each social grade in the particular supermarket as weights. Similarly, a ‘shopper mean age’ variable for each supermarket was calculated using the mid-range age within each age group and the proportion of grocery spend by each age group in the supermarket as weights.

### Data analysis

In the longitudinal analysis, controlled interrupted time series models were fitted [23,24], and we report results according to the recommendations described by Jandoc and colleagues [25]. Interrupted time series models involve plotting a regression line of the outcome grouped by time (here, purchases of common checkout foods per 4 weeks) against time. The slope of the preintervention trend is then carried forward after the ‘interruption’ (here, implementation of checkout food policies) as the ‘counterfactual’ of what was expected to happen had the intervention not occurred and compared to a similar regression line calculated from observations of what did happen following the interruption. Estimates of both the change in ‘level’ and ‘trend’ of the observed versus counterfactual regression lines are calculated. The level change is the difference in intercepts between regression lines estimated from observations before and after the interruption, whereas the trend change is the difference in slopes. Including a comparator group allows stronger estimation of the counterfactual of what would have been expected to happen had the intervention not occurred—from both the preimplementation data in the intervention case and the difference in preimplementation curves between intervention and comparator. The point of comparison is this counterfactual of what would have been expected to happen had the intervention not occurred.

We explored the association between implementation of checkout food policies and purchases of common checkout food over the period from 13 four-weekly periods (12 months) before implementation to 13 four-weekly periods (12 months) after implementation. As the intervention supermarkets have different customer bases, all implemented checkout food policies at different time points; and because different comparators were appropriate for each intervention supermarket (see below), we conducted separate analyses for each intervention supermarket. Aggregating data at the four-weekly period provided an optimal balance between a time period that was short enough to provide an adequate number of data points over 1 year to meet the minimum data requirements of interrupted time series analysis [24] and long enough to avoid unnecessary point-to-point ‘noise’.

Most supermarkets showed marked seasonal variations in purchases of foods commonly displayed at checkouts, with purchases appearing to peak around Valentine’s Day, Easter, and Christmas. Using a control group in interrupted time series models reduces the impact of such time-varying confounding [23]. A suitable control group should show a similar preimplementation curve, although not necessarily at the same level. Of the three comparison supermarkets available, two had a checkout food policy throughout the study period, and one had no policy throughout. This provided four comparison options: either of the three individually or a mean of the three. In the main analyses, we selected from these four options based on the visual similarity of preimplementation purchase curves. For one of the intervention supermarkets, none of the comparison options had similar preintervention purchase curves, and an uncontrolled model was used. To explore the sensitivity of our findings to the comparison group selected, we repeated all analyses using the mean values of the three comparison supermarkets throughout.

We adjusted models for four-weekly period (13 periods per year, so 12 indicator variables), Easter (because unlike other celebrations, its date varies from year to year; included as an indicator variable if the date fell in different four-weekly periods between years), and market share. Alternatives to four-weekly period indicator variables were considered to adjust for season, but none improved model fit.

Generalised least squares regression models were used assuming a normally distributed outcome and allowing for autoregressive and moving average correlation structures as appropriate. For each model, the fits of different autoregressive-moving-average models were compared with likelihood-ratio tests. Final models were chosen based on lower values of the Akaike information criterion (AIC) and Bayesian information criterion (BIC), as well as by visually assessing plots (see S1 Table). More parsimonious models were preferred when possible. To account for differences in market share between supermarkets and for any changes in these over time, estimated values in the 4 weeks immediately following implementation and the 12 months after implementation were divided by the relevant market share of each supermarket at the relevant point in time. Thus, the outcome in these analyses is units of common checkout foods purchased per 4 weeks per percentage market share. This allowed any change in custom associated with interventions to be taken into account and changes associated with implementation of checkout food policies in supermarkets with different markets shares to be more comparable.

The generalised linear models took the general form of

$$\begin{array}{l} {\text{Purchases}}_{fi}\sim \;\beta_{f}Trend_{f}+\beta_{i}Intervention_{i}+\;\beta_{fi}TrendIntervention_{fi}+\beta_{f}Policy_{f}\\ +\beta_{f}PolicyTrend_{f}+\beta_{fi}PolicyIntervention_{fi}\\ +\beta_{fi}TrendInteventionPolicy_{fi}+\;\beta_{t}Marketshare_{t}+\;\beta_{f}Easter_{f}+\beta_{f}Fourweek_{f}+\varepsilon_{fi} \end{array}$$

where *Purchases* denotes four-weekly unit purchases of common checkout foods, *Trend* denotes the overall four-weekly linear trend, *Intervention* denotes the indicator for intervention versus comparator supermarket, *TrendIntervention* denotes the intervention supermarket-specific four-weekly linear trend, *Policy* denotes an indicator for the period after checkout food policy implementation, *PolicyTrend* denotes the four-weekly linear trend after policy implementation, *PolicyIntervention* denotes the intervention supermarket-specific indicator for the period after checkout food policy implementation, *TrendInterventionPolicy* denotes the intervention supermarket-specific four-weekly linear trend after checkout food policy implementation, *Marketshare* denotes the 12-weekly percentage market share, *Easter* denotes an indicator for Easter, *Fourweek* denotes the vector of four-weekly period indicators, and *ε* represents the error term. The subscript *f* corresponds to four-weekly-specific variables (1–26), and *i* corresponds to intervention-specific variables.

Absolute and percentage differences between observed and counterfactual values in the 4-week period immediately after and 12 months after policy implementation were derived from the models. We used random effects meta-analysis [26] to synthesize these differences at 4 weeks and 12 months from different supermarkets. With only six intervention supermarkets in the meta-analyses, it was not possible to robustly separate by policy category (clear and consistent versus vague or inconsistent). The weights from the meta-analyses of the absolute differences were used to calculate weighted average percentage changes.

In the cross-sectional analysis, the association of checkout food policies (clear and consistent, vague or inconsistent, or no policy) with annual purchases of common checkout foods was estimated using a linear regression model, including supermarket group as a random effect. Here, the outcome variable was annual purchases of common checkout foods weighted and uplifted by Kantar, divided by annual market share, and log transformed, where appropriate. The exposure variable was checkout food policy type. The model was adjusted for year, ‘shopper mean age’, and ‘shopper mean social grade’. Log-transformed results were back converted to ratios for presentation and interpretation.

Data preparation, cross-sectional analyses, and meta-analyses were performed using Stata/SE v14.2 [27]. Interrupted time series models were fitted using R v3.3.1 [28].

### Ethics, research governance, and data sharing

Ethical approval was not required for these analyses of aggregate anonymised purchase data. Our commercial agreement with Kantar does not permit us to share data. All data are available to other researchers directly from Kantar (see www.kantarworldpanel.com/en for contact details).

The prospectively written proposal is included as a supporting information file (see S1 Text). The only deviation to the original proposal was additional inclusion of the cross-sectional data, which we were not aware of at the time of writing the proposal but which we feel add substantial strength to the analyses.

This paper is written in accordance with the STROBE statement (see S2 Table).

## Results

### Longitudinal analyses

Table 1 summarises the data used in the longitudinal analyses. Of nine included supermarkets, six implemented a new checkout food policy during the study period, three of which were clear and consistent and three of which were vague or inconsistent. The remaining three supermarkets did not implement new checkout food policies during the study period and were used as comparators. Two of these introduced vague or inconsistent policies before the study period began, and one had no policy throughout. Median 12-weekly supermarket market share varied from 3.1% (supermarkets 1 and 5) to 28.7% (supermarket 2). Median four-weekly purchases of common checkout foods per supermarket varied from 2,517,000 to 29,045,000 units.

**Table 1:** Description of included supermarkets and data in longitudinal analyses.

Supermarket	Checkout food policy type	Intervention or comparator	12-weekly market share (%), median [IQR]		Purchases of common checkout foods (1,000s of units/4 week), median [IQR]	
			Preimplementation	Post implementation	Preimplementation	Post implementation
1	Clear and consistent	Intervention	3.0 [2.9–3.1]	3.6 [3.5–3.6]	4,003 [3,748–4,425]	4,539 [3,585–5,151]
2	Clear and consistent	Intervention	28.9 [28.8–29.1]	28.4 [28.2–28.6]	27,998 [25,438–29,364]	28,336 [27,239–31,126]
3	Clear and consistent	Intervention	4.8 [4.6–4.8]	5.5 [5.4–5.6]	4,778 [4,386–5,525]	4,716 [4,364–5,151]
4	Vague or inconsistent	Intervention	4.9 [4.8–5.0]	5.1 [5.1–5.2]	2,851 [2,274–3,363]	3,247 [2,927–3,350]
5	Vague or inconsistent	Intervention	3.1^a^	3.1^a^	2,306 [2,36–2,661]	2,644 [2,343–2,800]
6	Vague or inconsistent	Intervention	10.9 [10.8–11.0]	10.6 [10.5–10.8]	10,531 [9,690–11,561]	10,678 [9,902–11,462]
7	Vague or inconsistent	Comparator	NA	16.9 [16.2–17.2]	NA	15,591.5 [13,288–16,656]
8	Vague or inconsistent	Comparator	NA	16.5 [16.4–16.8]	NA	12,241 [10,652–14,212]
9	Absent	Comparator	6.2 [6.1–6.4]	NA	10,221 [9,37–10,918]	NA

^a^Estimated from 2017 annual reports.

Abbreviations: IQR, interquartile range; NA, not applicable.

**Fig 2:**
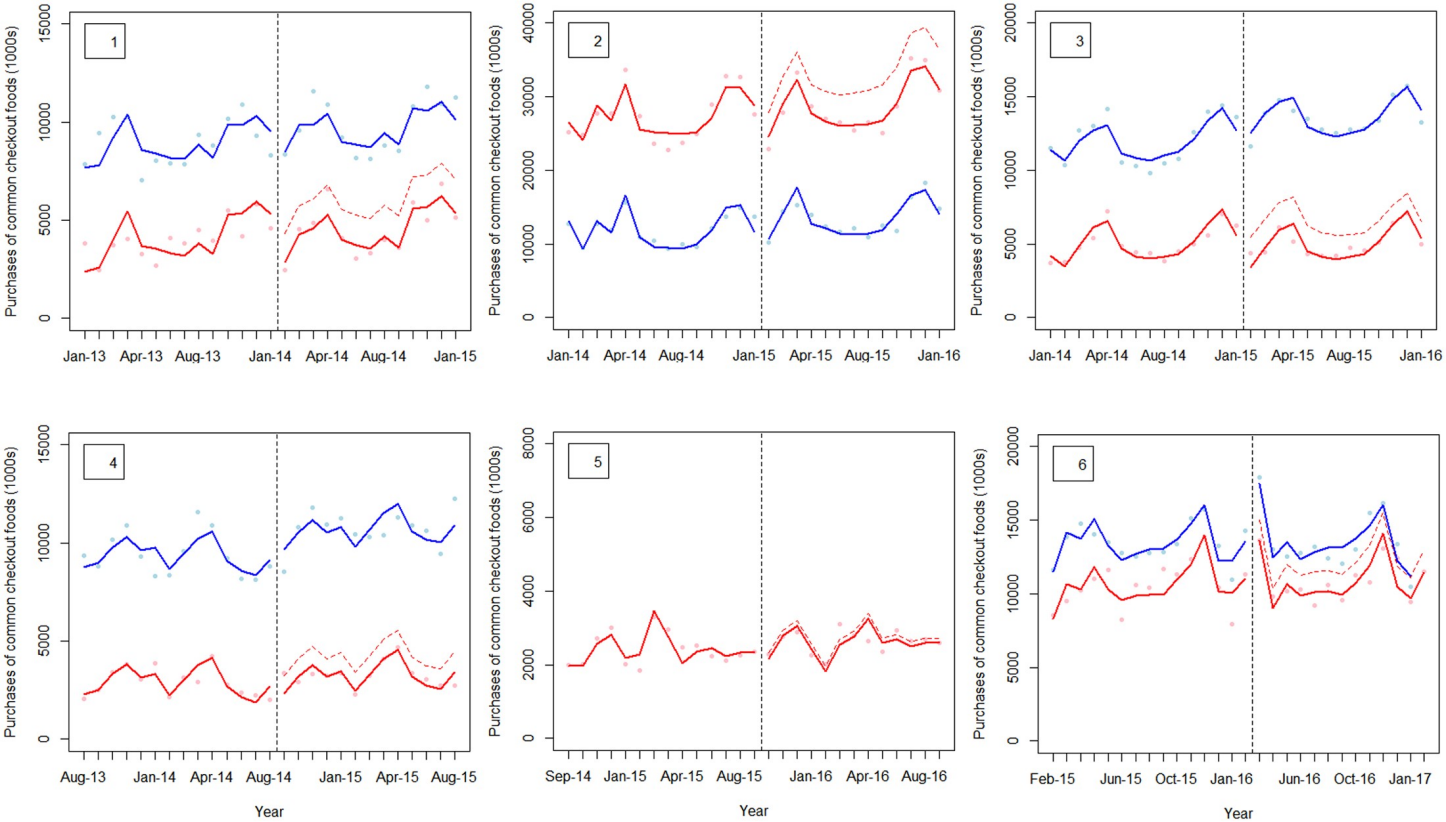
Interrupted time series models: Association between checkout food policy implementation and purchases of common checkout foods. ‘Best fit’ comparison group. Panel number indicates intervention store number as used elsewhere. Vertical black dotted line = time of implementation. Red line = intervention store, red dotted line = counterfactual, blue line = comparison store.

Table 2 summarises the results from the interrupted time series analysis models for each supermarket, showing estimates of immediate level and trend change as well as differences in purchases at 12 months. Fig 2 shows trends in purchases of common checkout foods for each pair of intervention and comparator from 12 months before to 12 months after implementation.

**Table 2:** Summary of the longitudinal associations between implementation of checkout food policies and purchases of common checkout foods, estimated from interrupted time series models (best-fit comparison group).

Intervention supermarket	1,000s of units of common checkout food purchases per % market share per 4 weeks (95% CI)					
	1	2	3	4	5	6
Comparison supermarket	9	8	Mean of 7, 8, 9	9	None	Mean of 7, 8, 9
Level change (4 weeks)	−461.6 (−1,016.3 to 93.1)	−108.7 (−137.1 to −80.4)	−435.8 (−690.0 to −181.5)	−178.7 (−294.0 to −63.3)	−54.2 (−274.8 to 166.4)	−118.1 (−326.9 to 90.7)
Trend change	−6.8 (−25.0 to 11.5)	−6.1 (−10.3 to −1.9)	14.8 (−11.4 to 41.0)	−2.7 (−14.2 to 8.8)	1.3 (−12.8 to 15.4)	−3.1 (−32.1 to 25.9)
Change (at 12 months)	−483.6 (−994.2 to 21.1)	−194.0 (−270.9 to −117.1)	−212.2 (−549.0 to 124.6)	−205.7 (−370.4 to −40.9)	−37.3 (−243.4 to 168.9)	−158.3 (−538.3 to 221.6)

All models are adjusted for market share, four-weekly time point, and Easter (if Easter fell in different four-weekly periods between years).

Abbreviation: CI, confidence interval.

Of three supermarkets that implemented clear and consistent policies (supermarkets 1, 2, and 3), there was a statistically significant immediate reduction (negative level change) in purchases of common checkout foods compared to the counterfactual associated with implementation policies in two cases (supermarkets 2 and 3). This was associated with a statistically significant change in trend and change in purchases at 12 months in supermarket 2 only.

Of three instances when a vague or inconsistent checkout food policy was introduced (supermarkets 4, 5, and 6), this was associated with a statistically significant immediate reduction in purchases of common checkout foods compared to the counterfactual in only one supermarket (supermarket 4). However, this change was sustained at 12 months. In the other two instances, no significant changes in level or trend of purchases of common checkout foods compared to the counterfactual were associated with the introduction of checkout food policies (supermarkets 5 and 6).

**Fig 3:**
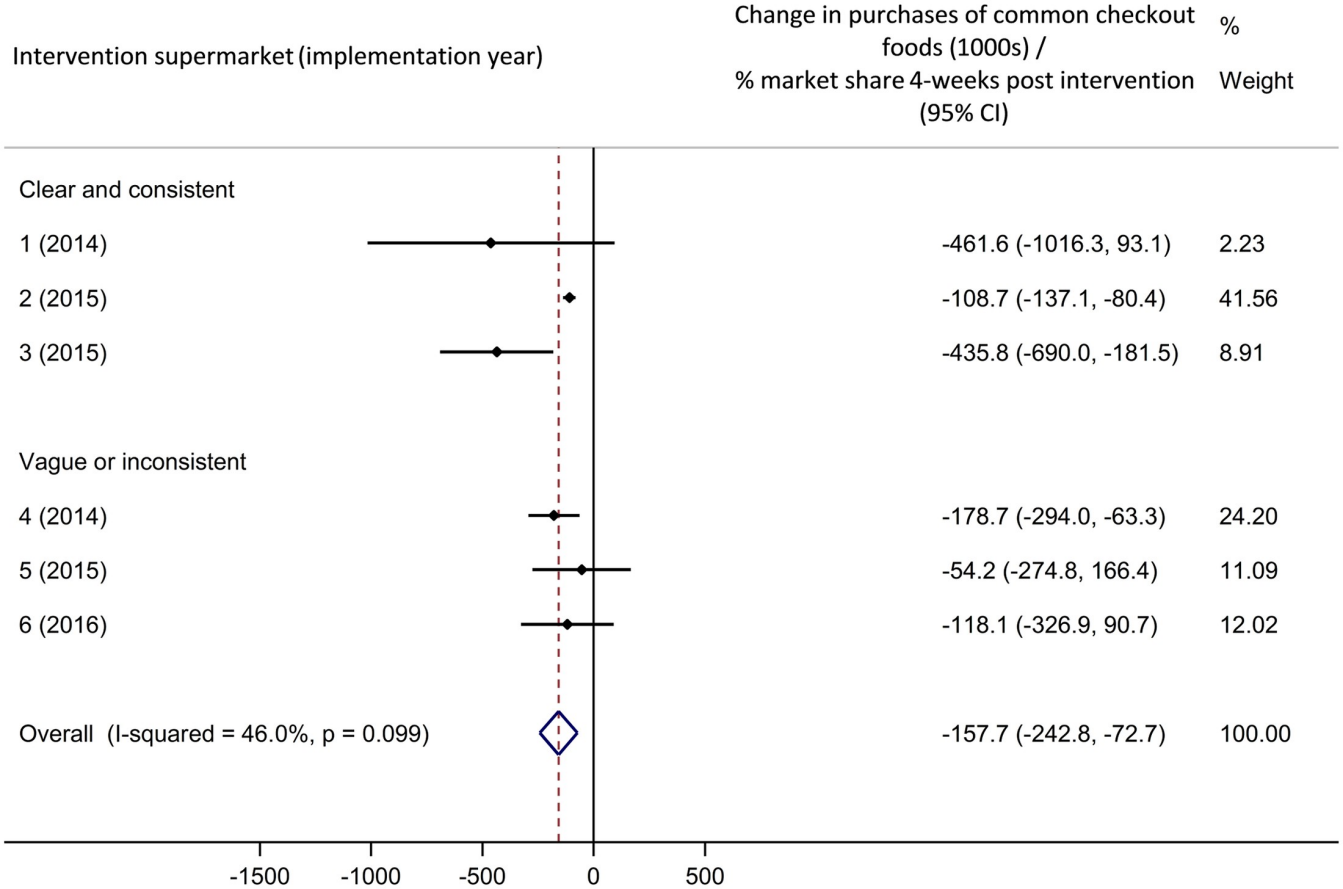
Meta-analysis: Association between checkout food policy implementation and purchases of common checkout foods, 4 weeks. ‘Best fit’ comparison group. CI, confidence interval.

**Fig 4:**
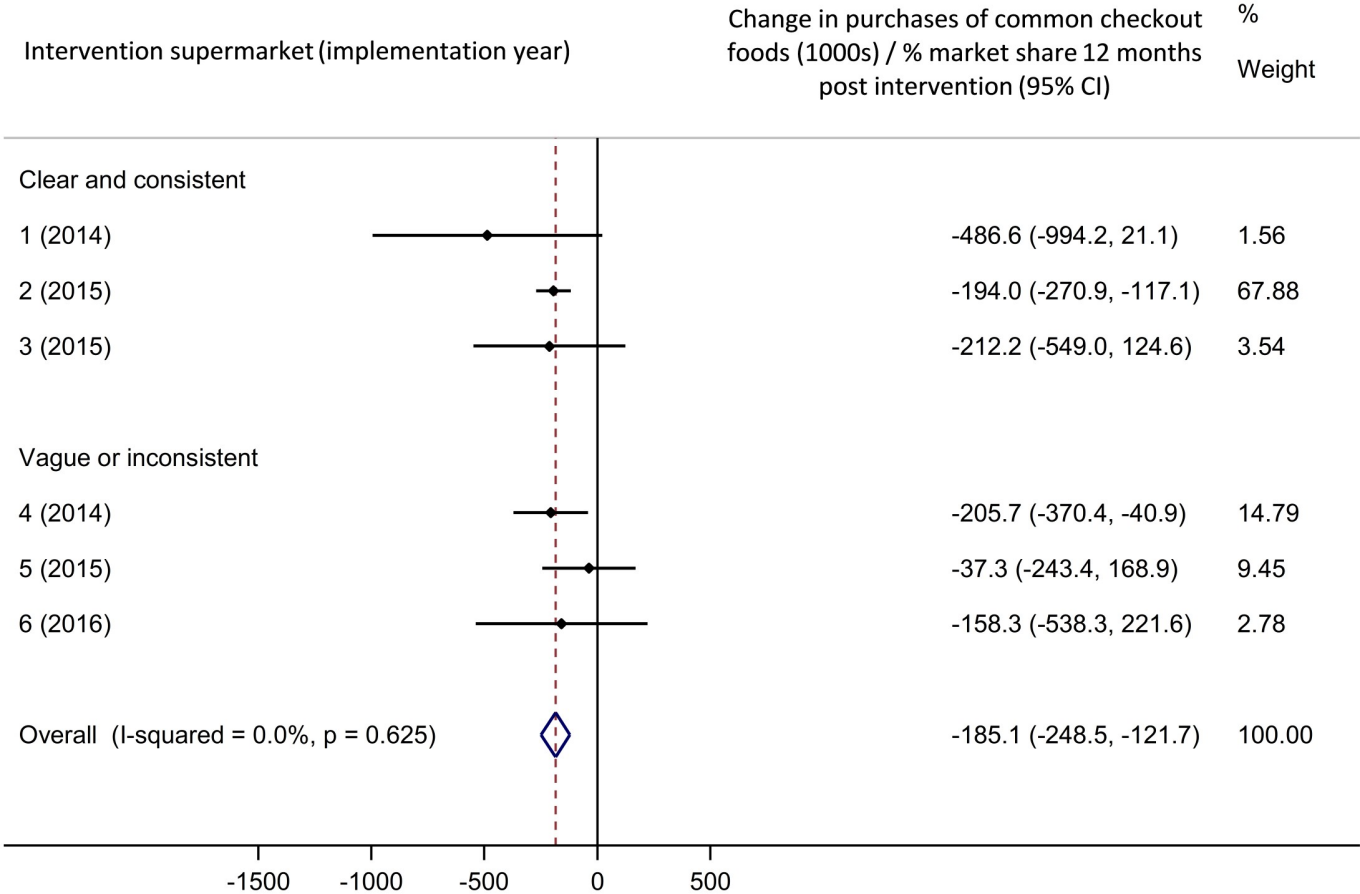
Meta-analysis: Association between checkout food policy implementation and purchases of common checkout foods, 12 months. ‘Best fit’ comparison group. CI, confidence interval.

Meta-analyses of these results are shown in Figs 3 and 4. Overall, implementation of supermarket checkout food policies was associated with a statistically significant decrease in purchases of common checkout foods of 157,700 packages per percentage market share in the 4 weeks following policy implementation (95% confidence interval [CI] 72,700–242,800 packages decrease, Fig 3) compared to the counterfactual. This effect was sustained at 12 months post implementation (Fig 4; 185,100 packages decrease per percentage market share [95% CI 121,700–248,500]). The weighted average percentage change in purchases compared to the counterfactual was −17.3% in the 4 weeks following implementation and −15.5% after 12 months.

The sensitivity analysis using mean values for the three comparison supermarkets for all analyses revealed a similar pattern of results to those described here (see S3 Table, S1, S2 and S3 Figs), except that the effect was diminished and no longer significant 12 months after implementation.

### Cross-sectional analyses

Table 3 describes market share and purchase data used in the cross-sectional analysis. As before, for each supermarket, year-to-year variation in market share was modest, but year-to-year variation in purchases of common checkout foods was more pronounced.

**Table 3:** Description of data used in cross-sectional analysis.

Supermarket group	Checkout food policy category	UK grocery market share (%)		Annual unit purchases of common checkout foods (1,000s)	
		2016	2017	2016	2017
1	Clear and consistent	4.3	4.7	15,203	18,752
2	Clear and consistent	28	27.5	218,200	247,600
3	Clear and consistent	6.0	7.0	16,313	14,585
4	Vague or inconsistent	5.0	4.7	17,066	14,870
5	Vague or inconsistent	3.1^a^	3.1^a^	30,603	34,097
6	Vague or inconsistent	10.3	10.0	90,969	54,069
7	Vague or inconsistent	15.0	15.0	100,100	114,700
8a	Vague or inconsistent	15.5	14.9	53,484	48,554
8b	Absent	0.8	0.9	33,787	34,029
9	Absent	6.2	6.0	94,652	85,991
Total	-	94.2	93.8	670,377	667,247

^a^Estimated from 2017 annual reports.

The linear regression model used in the cross-sectional analysis is summarised in Table 4. There were statistically significant fewer annual unit purchases of common checkout foods per percentage market share from supermarkets with checkout food policies than from supermarkets with no checkout food policy. The absolute differences reported in Table 4 are comparable to a 75.3% (95% CI 45.4%–88.8%) reduction in supermarkets with vague or inconsistent policies compared to none and a 79.5% (95% CI 44.7%–92.4%) reduction in supermarkets with clear and consistent policies versus none. There was no evidence of difference in purchases by policy category (*p* = 0.61), year, supermarket mean age, or social grade.

**Table 4:** Summary of the cross-sectional association between presence and nature of checkout food policies and purchases of common checkout foods, estimated from linear regression models.

Variable	Level	Annual unit purchases of common checkout food per percent market share in 1,000s (95% CI)
Checkout food policy status	None	Reference
	Vague or inconsistent	−22,400 (−32,100 to −12,700)
	Clear and consistent	−25,000 (−37,100 to −12,900)
Year	2016	Reference
	2017	−610 (−1,733 to 514)
Supermarket mean age	--	−840 (−2,896 to 1,216)
Supermarket mean social grade	--	11,800 (−9,317 to 33,000)

Abbreviation: CI, confidence interval.

## Discussion

This is the first study we are aware of to evaluate the impact of voluntary supermarket-led checkout food policies on purchases. It contributes to the small but growing literature on industry-led activities to promote healthier diets [29–33]. We conducted a pragmatic natural experimental evaluation of the introduction of checkout food policies in six of nine large UK supermarket chains, using two different but complementary data sources. In a longitudinal analysis of purchases brought into the home, we found that implementation of a checkout food policy was associated with a significant immediate reduction in purchases of single-serve or small packages of sugary confectionery, chocolate, and crisps of 17.3%. One year post implementation, the weighted average percentage change was 15.5% lower than what would have been expected if a checkout food policy had not been implemented. The immediate, but not sustained, effect was robust in the sensitivity analysis. In the cross-sectional analysis of purchases consumed ‘on the go’ in 2016 and 2017, we found 75.3%–79.5% fewer purchases of common checkout foods from supermarkets with versus without checkout food policies, supporting a longer-term effect of supermarket checkout food policies, especially for purchases eaten without being brought home. The cross-sectional analysis showed no difference between supermarkets with clear and consistent versus vague or inconsistent checkout food policies. We were not able to robustly formally test any difference by policy category in the longitudinal analyses.

### Strengths and weaknesses

The controlled, interrupted time series approach used in our longitudinal analyses is one of the strongest quasi-experimental designs available [25]. In longitudinal analyses, we used purchase data from a large, broadly representative consumer panel. As far as we are aware, this is the most comprehensive data on UK food purchases available across the full year and at the product level. As data are multiplied up and weighted to represent all UK households, our results are likely to be generalisable to the UK. However, retailing environments vary internationally [7], and our results may not be more widely generalisable.

In longitudinal analyses, we took into account secular trends and seasonal variations in purchases. Including comparison groups reduced the likelihood that the results are due to wider changes to the grocery market.

Purchases that never enter the home are not recorded by the take-home panel. This was addressed by additional cross-sectional analyses of on-the-go purchases. The on-the-go panel is smaller than the take-home panel and only recently established, precluding longitudinal analyses. However, triangulating different data and analyses lends robustness to natural experimental evaluations [18].

By focusing on supermarket purchases, we were unable to account for purchases displaced to non-supermarket locations—a previously reported response to supermarket interventions [34]. By expressing our longitudinal results as units purchased per percentage market share, we are able to take account of any overall movement of shoppers between supermarkets. By adjusting for mean shopper characteristics in the cross-sectional analyses, we determined that results are independent of who shops at different supermarkets. However, there may have been selective movement of particular types of shoppers associated with implementation of interventions that we were not able to take account of. Nor did we have information on where in the store purchases were selected. We restricted our analyses to smaller, single-serve package sizes to increase the likelihood that purchases were from checkouts. However, this may be neither sensitive nor specific. As we do not have information on purchases of sugary confectionery, chocolate, and crisps in larger package sizes, we also do not know if purchases were displaced from smaller to larger packages. This could be an area of future research. We have assumed that all supermarkets are comparable. However, some supermarket groups include larger proportions of convenience compared to larger stores. There may be greater sales of smaller package sizes from convenience stores.

The data we had access to combined purchases from all formats within each supermarket group in the longitudinal analysis. It is possible that the association between checkout food policies and purchases of common checkout food varies by format and that an interaction exists. Future research could explore this possibility.

As the data were retrospectively obtained, we were not able to take into account potential store-specific simultaneous interventions that might have taken place during the study period, which may have influenced either the longitudinal or cross-sectional results. Supermarkets are, however, continuously innovating. By adding comparison groups to the interrupted time series analyses and synthesising results from multiple supermarkets that introduced checkout food policies at different time points, we reduced the potential impact of wider marketing trends and changing consumer preferences on our results. However, we did not include a non-checkout food as a comparator.

Both our longitudinal and cross-sectional analyses were observational, and causality cannot be ascribed. Purchases do not necessarily represent consumption or reflect the composition of total diet.

### Comparison of findings to previous studies

There is some research describing foods displayed at supermarket checkouts [6–10,15,17]. However, we are not aware of previous evaluations of the impact of supermarket-led checkout food policies on purchases. Our previous survey found that supermarkets with clear and consistent checkout food policies were less likely to display any food, and a lower proportion of the food they did display was less-healthy, as defined by the Food Standards Agency’s Nutrient Profiling Model [15,16]. Furthermore, in one city, we found no difference in the healthfulness of food displayed near but not at checkouts according to checkout food policy [17]. These differences in food displayed at and near checkouts according to checkout food policy status may help to explain the differences in purchases reported here.

A range of researcher-led intervention studies focusing on or including healthier checkout foods have been conducted [35–39]. These report mixed findings, likely because of variations in the foods targeted and the level of implementation achieved. In contrast, we previously found a high level of adherence in those supermarkets with clear and consistent checkout food policies [15]. This may reflect supermarkets’ commitment to make their own policies work and their ability to respond adaptively to customer behaviour following implementation. For example, many different healthier checkout foods may be trialled before the optimum mix is identified.

In contrast to previous researcher-led interventions, with a maximum follow-up of 6 months [35–39], we included a follow-up period of 12 months in the longitudinal analyses. This allowed us to account for seasonal variations and to determine that short-term changes in purchases associated with checkout food policy implementation are not necessarily sustained at 12 months. The cross-sectional analysis allowed us to explore even longer-term effects. Given that we found a difference in purchases that was robust to sensitivity analysis immediately following implementation but not at 12 months, future research should include at least a 12-month follow-up to determine if any early changes are sustained.

### Interpretation and implication of findings

In longitudinal analyses, the implementation of checkout food policies was associated with an immediate decrease in take-home purchases of common checkout foods. Although this was sustained at 12 months in the main analysis, this was not robust in the sensitivity analysis. In cross-sectional analyses using data from 2016 to 2017, on-the-go purchases were significantly lower from supermarkets with versus without checkout food policies. Take-home purchases may more often be planned [40], whereas on-the-go purchases may more often be impulsive [2,8]. Our findings indicate that there is a potential impact of checkout food polices on both impulse and planned purchases.

In our synthesis of longitudinal analyses, we found a statistically significant association between implementation of checkout food policies and purchases. There was some indication that the effect was more pronounced in supermarkets with clear and consistent, compared to vague or inconsistent, policies. The same trend was seen at 12 months post implementation. Neither of these differences was formally tested, because of small numbers; however, this trend may reflect our previous findings of greater adherence to clear and consistent, compared to vague or inconsistent, checkout food polices [15]. Furthermore, research in more-controlled settings has shown that the balance of healthier foods displayed at checkouts influences customers’ behaviour, with healthier foods being more likely to be selected when they were in the majority [41]. In contrast, in cross-sectional analyses, there was no difference in the magnitude of effect between supermarkets with a clear and consistent checkout food policy compared to those with vague or inconsistent policies. This may reflect the differences in purchases included in the different analysis. ‘Take-home’ purchases captured in the longitudinal analyses may be differently influenced by different checkout food policies than ‘on-the-go’ purchases captured in the cross-sectional analysis.

Our findings indicate that supermarket-led activities can influence purchasing and hence may be an important vehicle to improve population health. In particular, our findings suggest checkout food policies may be one method to help decrease some purchases of less-healthy foods. Government-led nutritional standards on checkout food have been suggested [11,13] and may be attractive to retailers, as they provide a ‘level playing field’. Within supermarkets, several other interventions to promote healthier purchases have shown potential [42–45].

Food at checkouts has been found to lead to child purchasing requests [6] that parents can find hard to resist [9]. Anecdotal information from newspaper reports associated with the introduction of supermarket checkout food policies suggests that supermarkets implement these to improve the customer experience rather than to achieve any particular public health goals. That we previously found good adherence to policies [15] suggests that any change in purchases is outweighed by other benefits supermarkets gain from these policies.

### Unanswered questions and future research

Future research should prospectively explore how supermarkets and customers change their behaviour in response to supermarket-led interventions such as checkout food policies. Data on the effect of such policies on total diet would allow us to understand their potential for improving population health. Qualitative data exploring consumers’ views of the retail environment and supermarkets’ motivations for introducing policies may help to identify further opportunities for greater alignment between retail and public health policy. It would also be helpful to explore under what circumstances retailers, the public, and politicians would feel that further regulation in this area would be justified.

### Conclusions

The implementation of supermarket checkout food policies was associated with an immediate reduction in take-home purchases of sugary confectionery, chocolate, and crisps. There was some indication that this decrease was sustained 1 year post implementation, but this was not robust to sensitivity analysis. Data collected after implementation of all policies revealed that on-the-go purchases of common checkout foods appeared to be lower from supermarkets with versus without checkout food policies. Voluntary supermarket-led activities have the potential to promote healthier food purchasing.

## Supporting information

S1 Table Autoregressive and moving average correlational structures used in interrupted time series models. (DOCX)

S2 Table Completed STROBE checklist. (DOC)

S3 Table Summary of the association between implementation of checkout food policies and purchases of common checkout foods, estimated from interrupted time series models. Sensitivity analysis using mean values of the three comparison groups.
(DOCX)

S1 Fig Interrupted time series models: Association between checkout food policy implementation and purchases of common checkout foods. Sensitivity analysis using mean values of the three comparison groups.
(TIF)

S2 Fig Meta-analysis: Association between checkout food policy implementation and purchases of common checkout foods, 4 weeks. Sensitivity analysis using mean values of the three comparison groups.
(TIF)

S3 Fig Meta-analysis: Association between checkout food policy implementation and purchases of common checkout foods, 12 months. Sensitivity analysis using mean values of the three comparison groups.
(TIF)

S1 Text Original proposal. (DOCX)

## Abbreviations

AIC: Akaike information criterion
BIC: Bayesian information criterion
CI: confidence interval
IQR: interquartile range
