# Supplementary material for: Supermarket policies on less-healthy food at checkouts: Natural experimental evaluation using interrupted time series analyses of purchases
Source: PLoS Med. 2018 Dec 18;15(12):e1002712. doi: 10.1371/journal.pmed.1002712 (PMC6298641; doi:10.1371/journal.pmed.1002712)
Supplement: S1 Table — (DOCX) [file pmed.1002712.s001.docx]

**S1 Table: Autoregressive and moving average correlational structures used in interrupted time series models**

| Supermarket | ‘Best fit’ comparison group | | Mean comparison group | |
| --- | --- | --- | --- | --- |
|  | Autoregressive lag (p) | Moving average lag (q) | Autoregressive lag (p) | Moving average lag (q) |
| 1 | 4 | 1 | 3 | 0 |
| 2 | 6 | 0 | 2 | 0 |
| 3 | 0 | 4 | 0 | 4 |
| 4 | 6 | 0 | 2 | 0 |
| 5 | 0 | 3 | 0 | 2 |
| 6 | 1 | 0 | 1 | 0 |
