# Supplementary material for: Supermarket policies on less-healthy food at checkouts: Natural experimental evaluation using interrupted time series analyses of purchases
Source: PLoS Med. 2018 Dec 18;15(12):e1002712. doi: 10.1371/journal.pmed.1002712 (PMC6298641; doi:10.1371/journal.pmed.1002712)
Supplement: S2 Table — (DOC) [file pmed.1002712.s002.doc]

**S2 Table:** Completed STROBE Checklist

|  | Item No | Recommendation |
| --- | --- | --- |
| **Title and abstract** | 1 | (*a*) Indicate the study’s design with a commonly used term in the title or the abstract  **“Natural experimental evaluation” and “interrupted time series” are mentioned in the title** |
|  |  | (*b*) Provide in the abstract an informative and balanced summary of what was done and what was found **Abstract** |
| Introduction | | |
| Background/rationale | 2 | Explain the scientific background and rationale for the investigation being reported. **Introduction** |
| Objectives | 3 | State specific objectives, including any prespecified hypotheses. **Last paragraph of introduction** |
| Methods | | |
| Study design | 4 | Present key elements of study design early in the paper **First paragraph of methods.** |
| Setting | 5 | Describe the setting, locations, and relevant dates, including periods of recruitment, exposure, follow-up, and data collection **Methods.** |
| Participants | 6 | (*a*) Give the eligibility criteria, and the sources and methods of selection of participants. Describe methods of follow-up. **Eligibility of supermarkets described under “Supermarkets and checkout food policies”. Source of purchase data described under “Purchase data, market share, and demographic characteristics of customers”.** |
|  |  | (*b*) For matched studies, give matching criteria and number of exposed and unexposed. **Control supermarkets** **described under “Data analysis”.** |
| Variables | 7 | Clearly define all outcomes, exposures, predictors, potential confounders, and effect modifiers. Give diagnostic criteria, if applicable. **Outcomes described under “Definition of common checkout foods”. Exposures described under “Supermarkets and checkout food policies”. Potential confounders described under “Purchase data, market share, and demographic characteristics of customers”.** |
| Data sources/ measurement | 8* | For each variable of interest, give sources of data and details of methods of assessment (measurement). Describe comparability of assessment methods if there is more than one group. **Outcomes described under “Definition of common checkout foods”. Exposures described under “Supermarkets and checkout food policies”. Potential confounders described under “Purchase data, market share, and demographic characteristics of customers”.** |
| Bias | 9 | Describe any efforts to address potential sources of bias **Potential sources of bias addressed are supermarket market share (described under “Purchase data, market share, and demographic characteristics of customers”); co-interventions (addressed by including comparator supermarkets, described under “Data analysis”); and differential customer base (described under “Purchase data, market share, and demographic characteristics of customers”).** |
| Study size | 10 | Explain how the study size was arrived at. **Described under “Supermarkets and checkout food policies”.** |
| Quantitative variables | 11 | Explain how quantitative variables were handled in the analyses. If applicable, describe which groupings were chosen and why. **Supermarket groupings described under “Supermarkets and checkout food policies”. Demographic groupings described under “Purchase data, market share, and demographic characteristics of customers”.** |
| Statistical methods | 12 | (*a*) Describe all statistical methods, including those used to control for confounding **Described under “Data analysis”.** |
|  |  | (*b*) Describe any methods used to examine subgroups and interactions **No subgroup or interaction analyses are conducted.** |
|  |  | (*c*) Explain how missing data were addressed **There was no missing data.** |
|  |  | (*d*) If applicable, explain how loss to follow-up was addressed **There was no loss to follow-up.** |
|  |  | (*e*) Describe any sensitivity analyses **Described under “Data analysis”.** |
| Results | | |
| Participants | 13* | (a) Report numbers of individuals at each stage of study—eg numbers potentially eligible, examined for eligibility, confirmed eligible, included in the study, completing follow-up, and analysed. **Table 1 and Table 3.** |
|  |  | (b) Give reasons for non-participation at each stage. **There was no non-participation.** |
|  |  | (c) Consider use of a flow diagram **Figure 1.** |
| Descriptive data | 14* | (a) Give characteristics of study participants (eg demographic, clinical, social) and information on exposures and potential confounders. **Table 1 and 3.** |
|  |  | (b) Indicate number of participants with missing data for each variable of interest. **There was no missing data.** |
|  |  | (c) Summarise follow-up time (eg, average and total amount). **There was no follow up of individual participants. Follow up of supermarkets described in Figure 1.** |
| Outcome data | 15* | Report numbers of outcome events or summary measures over time. **Table 1 and Table 3.** |
| Main results | 16 | (*a*) Give unadjusted estimates and, if applicable, confounder-adjusted estimates and their precision (eg, 95% confidence interval). Make clear which confounders were adjusted for and why they were included. **Table 2.** |
|  |  | (*b*) Report category boundaries when continuous variables were categorized. **Not applicable.** |
|  |  | (*c*) If relevant, consider translating estimates of relative risk into absolute risk for a meaningful time period.  **Absolute changes presented in Table 2, Figure 2, Figure 3 and Table 4. Relative changes are reported in the text.** |
| Other analyses | 17 | Report other analyses done—eg analyses of subgroups and interactions, and sensitivity analyses. **Sensitivity analyses reported in S3 Table S3, S1 Figure, S2 Figure and S3 Figure.** |
| Discussion | | |
| Key results | 18 | Summarise key results with reference to study objectives. **Described in the first paragraph of the discussion.** |
| Limitations | 19 | Discuss limitations of the study, taking into account sources of potential bias or imprecision. Discuss both direction and magnitude of any potential bias. **Described under “Strengths and weaknesses”** |
| Interpretation | 20 | Give a cautious overall interpretation of results considering objectives, limitations, multiplicity of analyses, results from similar studies, and other relevant evidence. **Described under “Interpretation and implication of findings”** |
| Generalisability | 21 | Discuss the generalisability (external validity) of the study results. **Described under “Strengths and weaknesses”** |
| Other information | | |
| Funding | 22 | Give the source of funding and the role of the funders for the present study and, if applicable, for the original study on which the present article is based. **Provided in on-line submission system.** |

*Give information separately for exposed and unexposed groups.

**Note:** An Explanation and Elaboration article discusses each checklist item and gives methodological background and published examples of transparent reporting. The STROBE checklist is best used in conjunction with this article (freely available on the Web sites of PLoS Medicine at http://www.plosmedicine.org/, Annals of Internal Medicine at http://www.annals.org/, and Epidemiology at http://www.epidem.com/). Information on the STROBE Initiative is available at http://www.strobe-statement.org.
