# Supplementary material for: Supermarket policies on less-healthy food at checkouts: Natural experimental evaluation using interrupted time series analyses of purchases
Source: PLoS Med. 2018 Dec 18;15(12):e1002712. doi: 10.1371/journal.pmed.1002712 (PMC6298641; doi:10.1371/journal.pmed.1002712)
Supplement: S3 Table — Sensitivity analysis using mean values of the three comparison groups. (DOCX) [file pmed.1002712.s003.docx]

**S3 Table: Summary of the longitudinal associations between implementation of checkout food policies and purchases of common checkout foods, estimated from interrupted time series models (mean comparison group).**

|  | 1000s of units of common checkout food purchases per % market share per 4 weeks (95% confidence intervals) | | | | | |  |
| --- | --- | --- | --- | --- | --- | --- | --- |
| Intervention supermarket | 1 | 2 | 3 | 4 | 5 | 6 | |
| Comparison supermarket | Mean of 7,8,9 | Mean of 7,8,9 | Mean of 7,8,9 | Mean of 7,8,9 | Mean of 7,8,9 | Mean of 7,8,9 | |
| Level change (4 weeks) | -392.6 (-913.5;128.3) | -86.7 (-190.9;17.5) | -435.8 (-690.0;-181.5) | -201.3 (-469.4;66.7) | -529.4 (-935.7;-123.1) | -118.1 (-326.9;90.7) | |
| Trend change | -44.7 (109.4;20.0) | 5.1 (-10.4;20.7) | 14.8 (-11.4;12.7) | -18.3 (-50.7;14.1) | 65.4 (14.1;116.7) | -3.1 (-23.1;25.9) | |
| Change (at 12 months) | -862.5 (-1679.8;-45.3) | -20.5 (-235.8;194.8) | -212.2 (-549.0;124.6) | -421.6 (-856.8;13.7) | 320.9 (-228.6;870.5) | -158.3 (-538.3;221.6) | |

All models are adjusted for market share, 4-weekly time point, and Easter (if Easter fell in different 4-weekly periods between years).
