# Supplementary figures and images for: Supermarket policies on less-healthy food at checkouts: Natural experimental evaluation using interrupted time series analyses of purchases
Source: PLoS Med. 2018 Dec 18;15(12):e1002712. doi: 10.1371/journal.pmed.1002712 (PMC6298641; doi:10.1371/journal.pmed.1002712)

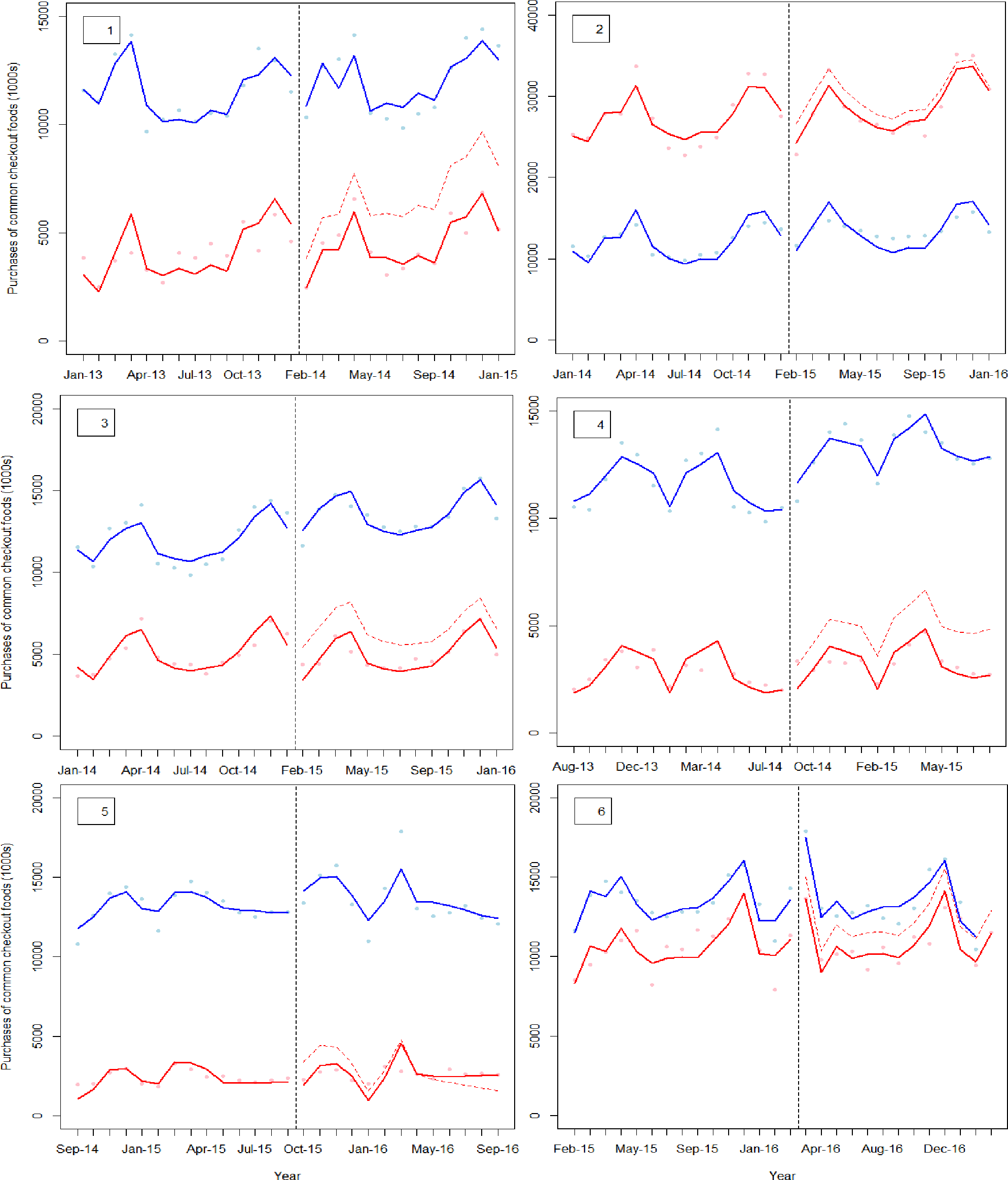

Supplement: S1 Fig — Sensitivity analysis using mean values of the three comparison groups. (TIF) [file pmed.1002712.s004.tif]

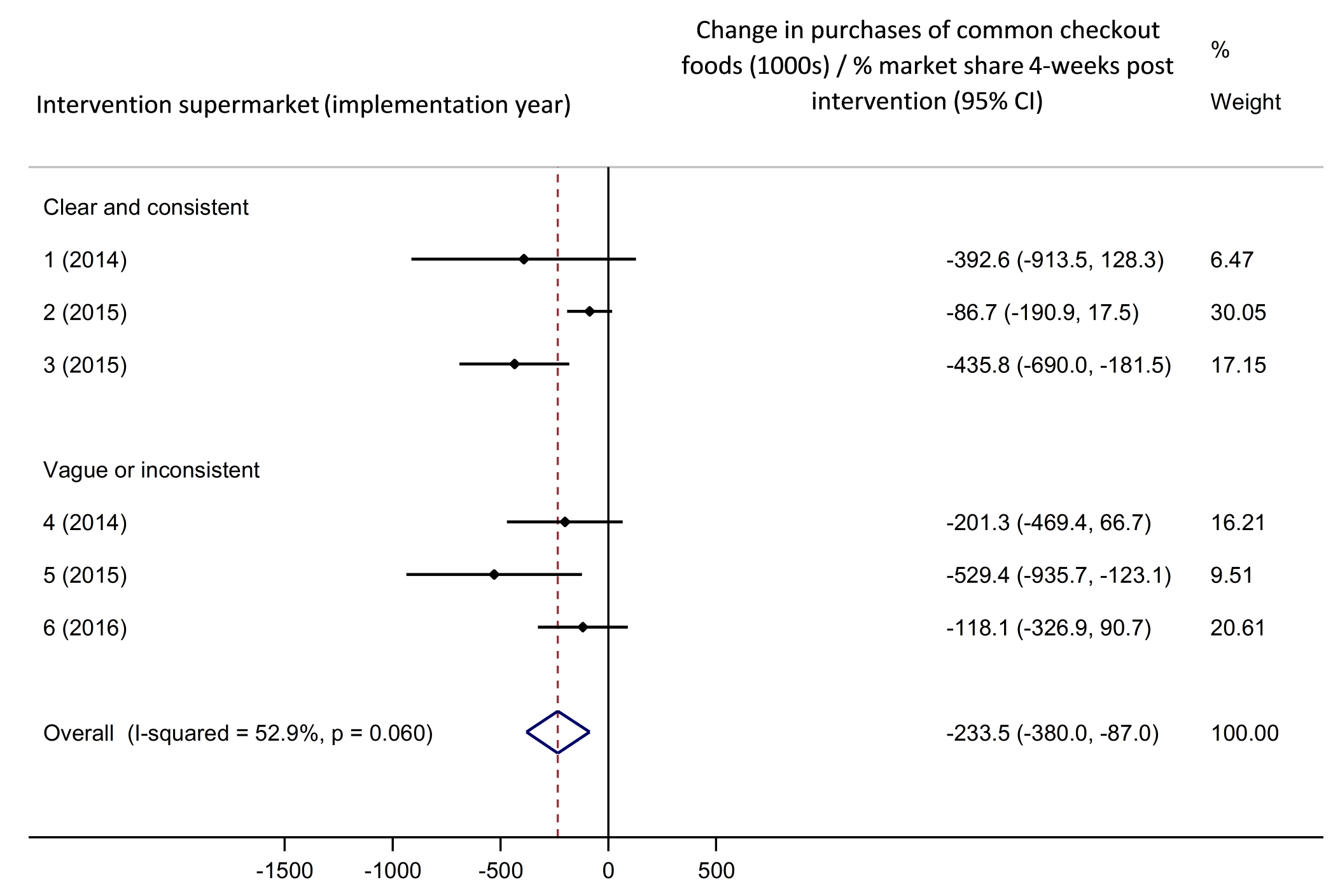

Supplement: S2 Fig — Sensitivity analysis using mean values of the three comparison groups. (TIF) [file pmed.1002712.s005.tif]

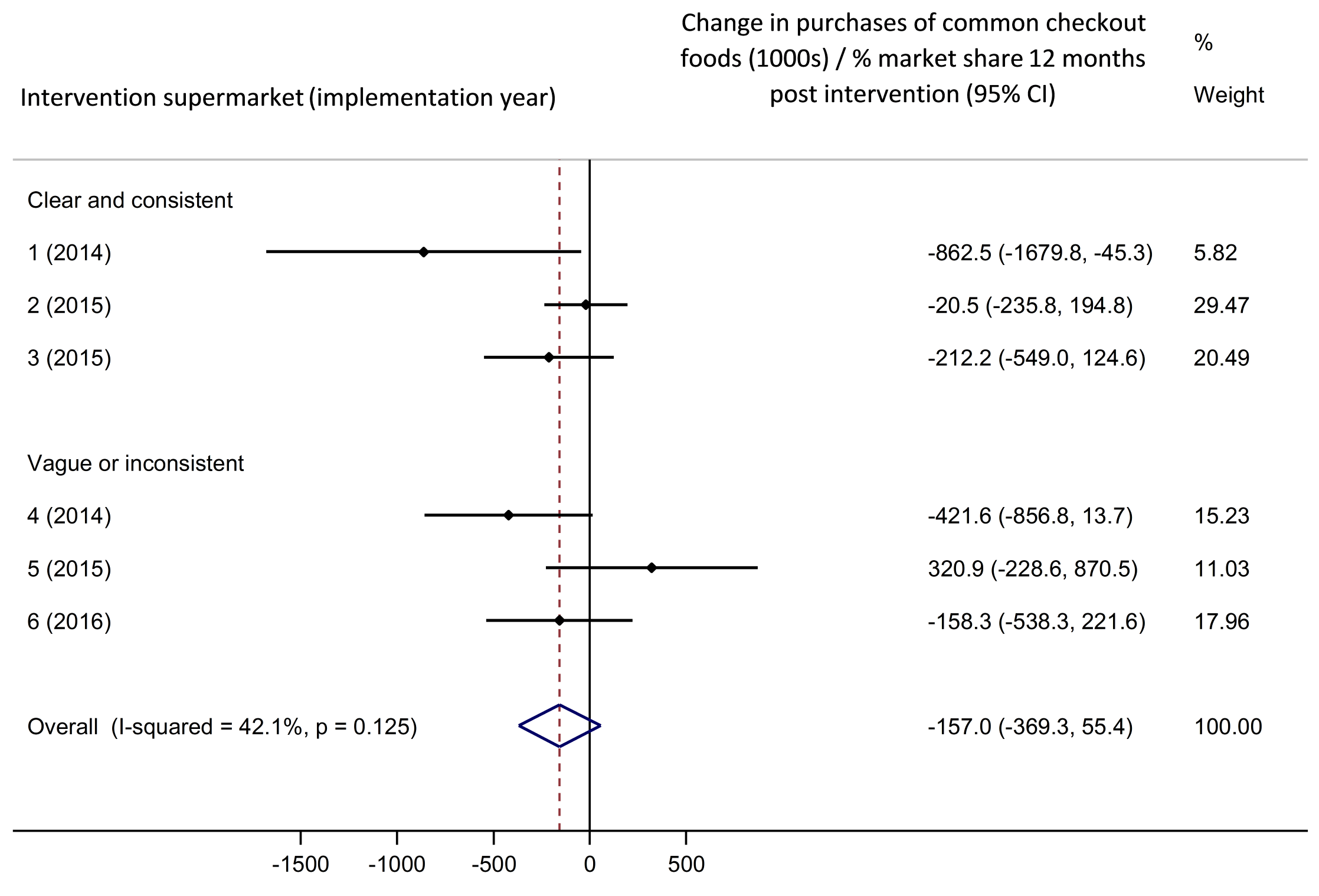

Supplement: S3 Fig — Sensitivity analysis using mean values of the three comparison groups. (TIF) [file pmed.1002712.s006.tif]
