# Supplementary material for: Supermarket policies on less-healthy food at checkouts: Natural experimental evaluation using interrupted time series analyses of purchases
Source: PLoS Med. 2018 Dec 18;15(12):e1002712. doi: 10.1371/journal.pmed.1002712 (PMC6298641; doi:10.1371/journal.pmed.1002712)
Supplement: S1 Text — (DOCX) [file pmed.1002712.s007.docx]

**DEPARTMENT OF HEALTH**

**PROJECT PROPOSAL**

| **A** | **SUMMARY OF PROJECT** | | | | | |
| --- | --- | --- | --- | --- | --- | --- |
| **1)** | **Principal investigator details:** | | | | | |
|  | *Name:* | Martin White | | | | |
|  | *Title:* | Professor | | | | |
|  | *Post held:* | Director of Research and Honorary Consultant in Public Health | | | | |
|  | *Institution:* | Centre for Diet & Activity Research (CEDAR), MRC Epidemiology Unit, University of Cambridge | | | | |
|  | *Address:* | Box 285 Institute of Metabolic Science, School of Clinical Medicine, Addenbrooke’s Hospital, Cambridge CB2 0QQ | | | | |
|  | *Email:* | [martin.white@mrc-epid.cam.ac.uk](mailto:martin.white@mrc-epid.cam.ac.uk) | | | | |
|  | *Phone:* | 01223 769159 | | | | |
| **2)** | **Project title:** | | | | | |
|  | **Exploring the impact of removing less healthy food from retail checkouts** | | | | | |
| **3)** | **Co-investigators:** | | | | | |
|  | *Name:* | | *Institution:* | | | |
|  | Dr Jean Adams | | Centre for Diet & Activity Research (CEDAR), MRC Epidemiology Unit, University of Cambridge | | | |
|  | Prof Ashley Adamson | | Institute of Health & Society, Newcastle University | | | |
|  | Dr Martine Stead | | Institute of Social Marketing, Stirling University | | | |
| **4)** | **Responsibilities of research team / Responsibility of each collaborator:** | | | | | |
|  | MW will be Co-PI, provide overall leadership for the research and lead in store observations and interviews with consumers and stakeholders. JA will be co-PI, provide day to day management of the research and lead quantitative analysis of sales data. AA will provide expertise in nutritional analysis. MS will provide expertise on qualitative research with consumers and stakeholders. All co-investigators will contribute to detailed study design, data interpretation and reporting. | | | | | |
| **5)** | **Abstract of project:** (200 words maximum) | | | | | |
|  | Background: | The display and promotion of less healthy food at supermarket checkouts has become commonplace, resulting in impulse purchases and child purchasing requests. A number of UK grocery retailers (e.g. Aldi, Lidl and Tesco) have recently committed to remove confectionary from checkouts in all their stores. The impact of such policies on food purchases have not been evaluated. Note: throughout this proposal we use the term ‘food’ to refer to all food and drink products including both non-alcoholic and alcoholic drinks. | | | | |
|  | Aims: | 1. Clarify the commitments of the major UK supermarkets to healthier checkouts; 2. Determine adherence of UK supermarket chains with healthier checkout food commitments to their commitments; and compare their alternative checkout products with those of UK supermarkets that have not yet made such commitments; 3. Compare the sales of common ‘less healthy’ checkout foods (identified in (2)), over time from different supermarket chains to explore if there is any impact of the introduction of healthier checkout food commitments on sales; 4. Assess whether any trends identified in (3) vary by age, gender or socio-economic position of customers; 5. Explore parents’ experiences of, and views on, promotion and placement of common checkout food and non-food products (identified in (2)); 6. Explore experiences relating to changing checkout food, and views on the challenges and opportunities for further action in this area amongst relevant senior stakeholders. | | | | |
|  | Methods: | We will use in-store observations across the eight leading supermarket chains, together with analysis of commercially available data on sales of products from different supermarkets, over time, before and after introduction of healthier checkout food commitments. Qualitative focus group discussions with parents and interviews with stakeholders will assess views on checkout sales and associated policies. | | | | |
| **6)** | **Coverage of consortium themes:** | | | | | |
|  | Health inequalities | | | ✓ | Smoking |  |
|  | Risk and health | | | ✓ | Obesity | ✓ |
|  | Incentives & regulation | | | ✓ | Work environment |  |
|  | Translation to policy | | | ✓ | Methodological development | ✓ |
| **7)** | **Policy relevance:** | | | | | |
|  | Removing less healthy food & drinks from checkouts is a simple voluntary intervention that seems to be acceptable to some supermarkets and forms the subject of current pressure group campaigns. However, the impact of healthier checkout food commitments on sales remains unknown; and public, political and commercial attitudes to these policies have not been clarified. | | | | | |
| **8)** | **Proposed start date and duration:** | | | | | |
|  | Start date: | 01.01.2017 | | | | |
|  | End date: | 30.02.2018 | | | | |
|  | Duration: | 13 months | | | | |
| **9)** | **Total cost: £186,319 (at 80% fEC); £232,898 (at 100% fEC)** | | | | | |

| **B** | **DETAILS OF PROJECT** |
| --- | --- |
| **1)** | **Scientific background and policy relevance:** |
|  | Exposure to energy dense, nutrient poor foods (including drinks) in everyday life contributes to the development of obesity.^1^ One potential source of such food, which has gained media,^2^ campaign group^3, 4^ and research^5-9^ attention is the display of energy dense, nutrient poor food at the checkouts of supermarkets and other, non-food, stores. One way of identifying these foods is using the Food Standards Agency nutrient profiling model, which dichotomises all foods and drinks into those that are high in salt, fat or sugar (HFSS, ‘less healthy’) and those that are not.^10^ Such foods are already subject to advertising restrictions on television in the UK.^11^  Across the developed world, supermarket checkout food tends to be less healthy and positioned to attract children.^5-9^ This phenomenon has also been documented in high street non-food stores.^12^ Checkout food leads to impulse purchases and child purchasing requests,^8, 13^ which parents find hard to resist.^4^ The balance of healthier to less healthy checkout food influences purchasing, with healthier foods being more likely to be selected when they dominate.^14^ As elsewhere, price promotions on checkout food increase sales and prompt impulse purchases.^15, 16^  In response to consumer concern, in 2014 two large UK grocery retailers (Tesco and Lidl) announced their intention to remove confectionary from checkouts in all stores, followed in 2015 by Aldi. Other supermarkets have introduced more limited policies. For example, Morrison’s has introduced ‘guilt free checkout lanes’ in some stores and M&S has introduced some healthier checkouts in some stores.  A large scoping review on retail micro-environments recently identified that changing the availability of healthy food can alter purchasing;^43, 44^ but no studies on checkout food were identified. It is not known what the effect of healthier checkout food commitments is on what checkout food (and other checkout products) is displayed or on purchases of common checkout food items from participating stores. |
| **2)** | **Purpose of research (including aims and objectives):** |
|  | The overall purpose of the research is to explore the impact of the introduction of healthier checkout food commitments in UK supermarkets on the availability and purchasing of common less healthy checkout foods (and other checkout products), and the experiences and perceptions of such policies amongst relevant stakeholders, including parents, policymakers and retailers. The objectives are to:   1. Clarify the commitments of the major UK supermarkets to healthier checkouts; 2. Determine adherence of UK supermarket chains with healthier checkout food commitments to their commitments; and compare their alternative checkout products with those of UK supermarkets that have not yet made such commitments; 3. Compare the sales of common ‘less healthy’ checkout foods (identified in (2)), over time from different supermarket chains to explore if there is any impact of the introduction of healthier checkout food commitments on sales; 4. Assess whether any trends identified in (3) vary by age, gender or socio-economic position of customers; 5. Explore parents’ experiences of, and views on, promotion and placement of common checkout food and non-food products (identified in (2)); 6. Explore experiences relating to changing checkout food, and views on the challenges and opportunities for further action in this area amongst relevant senior stakeholders. |
| **3)** | **Research plan:** |
|  | **Work package (WP) 1 - Cross-sectional comparison of checkout food availability in stores with healthier checkout policies and those without**  We will contact all major supermarket retailers to seek detailed information on their checkout food policies and any healthier checkout food commitments they have made.  We will conduct in-store observations of checkout food, and other products, using methods we have previously developed.^45^ By including both food and other products we will be able to explore what less healthy checkout food is replaced with whether it be healthier food or non-food products. Nine large national supermarket chains, representing more than 90% of UK grocery market-share, will be included: Tesco, Asda, Sainsbury’s, Morrison’s, Co-op, Aldi, Waitrose, Lidl, and M&S. Five shops in the East of England belonging to each chain, and representing all formats provided by the chain (e.g. out-of-town hypermarkets, in-town supermarkets, supermarket convenience stores formats) will be selected for inclusion using stratified random sampling from lists generated from supermarket web sites.  Observations will determine the range of food, and other, products available. As previously,^45^ we will define checkouts as any compulsory areas that shoppers have to pass through in order to pay for their goods. Many stores have shared queuing areas used for a number of payment points. In these cases, checkout areas will be considered as beginning in the shared main queuing area and finishing at the payment point. Products within arm’s reach of any point of the checkout area will be defined as checkout products. Products present immediately adjacent to the payment point will also be included. As previously,^45^ we will record the details of each product line available in checkout areas, not the volume of each line displayed. We will not seek permission from stores to make observations and, as such, believe it is inappropriate to take photographs to aid data recording. Instead, data will be recorded using discrete, mobile-phone type voice recorders with recordings transcribed soon after recording.  Checkout foods will be classified as less healthy or not^46^ and the proportion of checkout food in each store, format, and chain that is less healthy compared. We will also identify which foods meet the policy commitments of the supermarkets they are displayed in. In each store the location of the main and other displays (i.e. aisles) of confectionery and their proximity to, and visibility from, checkouts will also be recorded, so as to capture potential displacement of confectionery to elsewhere, and particularly displays visible (and easily reachable) from checkouts. Finally, we will explore any differences in our findings according to deprivation scores allocated to supermarkets according to their location at lower super output area level.  **WP2 - Longitudinal comparison of sales of foods commonly sold at checkouts across stores**  Data on overall sales of common checkout foods (identified in WP1), before and after implementation of healthier checkout food commitments, will be obtained from Kantar WorldPanel (a market research company). Data will be analysed using interrupted time-series analysis (ITSA) statistical methods.^17, 18^ Good practice guidance states that at least 10 data points before and after intervention implementation should be included in an ITSA.^18^ We will include monthly sales of common checkout foods for at least 12 months before and 12 months after introduction of healthier checkout commitments in each chain included in WP1 (Tesco, Asda, Sainsbury’s, Morrison’s, Co-op, Aldi, Waitrose, Lidl, M&S).  **WP3 - Distributional impacts of healthier checkout policies**  Building on WP2, we will explore any distributional effects of healthier checkout food commitments on sales of common checkout foods according to the age, sex and socio-economic positon of consumers. Data available from Kantar WorldPanel identifies the product purchased, and the supermarket chain from which the item is obtained, and demographic characteristics of the consumer. Using this data we will repeat the main analyses in WP2 to explore whether the introduction of healthier checkout food commitments impacted differentially on purchases of common checkout foods by consumers according to age, sex and socio-economic position.  **WP4 - Qualitative focus group discussions with parents**  We will undertake a series of focus group discussions with parents of primary school aged children (5-11 years) who are regular shoppers in the supermarket chains included in WP1 (Tesco, Asda, Sainsbury’s, Morrison’s, Co-op, Aldi, Waitrose, Lidl, M&S). Parents will be purposively sampled in order to achieve a maximum variation sample in relation to usual main food store (brand and format), whether they are a single parent and socio-economic position (based on social groups defined by the National Readership Survey – AB, C1C2, DE). We will commission a market research company to undertake sampling and hosting of focus groups on our behalf.  Discussions will be prompted by a topic guide and will seek to explore parents’ views and experiences of supermarket checkouts, the food and other goods for sale surrounding them, the engagement of their children with checkout food and other goods for sale, as well as in-store food promotions and supermarket shopping with children more generally. We will also seek parents’ views of voluntary and compulsory restrictions on in-store placement and promotion of foods, including healthier checkout food policies.  Focus groups will be facilitated and observed by our researcher, and digitally audio-recorded with written, informed consent of participants. Focus group discussions will be transcribed verbatim and analysed thematically using the Framework method.^19^ We will aim to undertake approximately 12 focus groups, each involving approximately 7-8 participants. Groups will be stratified according to single parent status and social group of participants. Thus, there will be 1-2 focus groups each containing participants with the following characteristics: single parents, social group AB; single parents, social groups C1C2; single parents, social groups DE; not single parents, social group AB, not single parents, social groups C1C2; not single parents, social groups DE.  **WP5 - Individual, qualitative telephone or face-to-face interviews with stakeholders**  We will undertake one-to-one interviews (n~25-30) with key stakeholders with an interest, or previous involvement, in healthier checkout policies. These will include: managers and strategists from the supermarket chains included in WP1 (Tesco, Asda, Sainsbury’s, Morrison’s, Co-op, Aldi, Waitrose, Lidl, M&S), representatives of supermarket trade and food retailing trade organisations, public health policymakers at local and national levels (e.g. from Department of Health, Public Health England, local authorities) and relevant civil society groups (e.g. Children’s Food Campaign, Food Foundation, UK Health forum). Interviews will be digitally audio-recorded with written, informed consent of participants, and transcribed verbatim. Data will be analysed thematically using the Framework method.^19^  **Ethics, governance and safety**  WP1-3 will not require ethical approval. No information on any individuals will be collected in WP1. WP2&3 will include analysis of aggregate sales data from which no individuals will be identifiable. All observational data will be anonymised as soon as possible after data collection and individual stores and chains will not be identifiable in any outputs.  The fieldworker who collects in-store data in WP1 will follow the MRC Epidemiology Unit’s lone worker policy, checking in at least daily with their line manager. Whilst they will not proactively identify themselves to store staff, they will carry formal university identification (university ID card) and a letter on headed paper describing who they are and what they are doing. They will produce these if challenged, be polite in all dealings and store staff and other customers, and leave stores if requested to at any point (if this occurs before data collection is complete, a replacement store will be selected for inclusion).  WP2-3 will be entirely desk based.  Ethical approval will be sought from the University of Cambridge, Department of Psychology Research Ethics Committee for WP4&5. Normal procedures will be followed for ensuring all participants give written informed consent before data collection begins, and that interviews and focus groups are respectful and inclusive. We will offer to reimburse participants’ for their time, travel and childcare expenses at a flat rate of £20 per participant.  Researchers collecting data for WP4 will always work in at least pairs meaning that lone working procedures will not be relevant. Data for WP5 will be collected via telephone interviews and hence will be desk based.  All research data will be stored in locked filing cabinets and on password protected servers and will be subject to local data security procedures at the Universities of Stirling and Cambridge as appropriate. Data from Stirling will be transferred to Cambridge via Secure File Transfer Protocol for archiving at the end of the project. All personally identifiable data will be stored separately from interview transcripts and other research data. All transcripts from focus groups and individual interviews will be anonymised as they are typed.  We will make data from WP1,4&5 available for sharing with other researchers via the MRC Epidemiology Unit website. As the data used in WP2&3 will be purchased from a commercial data provider, we do not expect to have permission to share this. |
| **4)** | **Links with providers/users (if not covered under research plan):** |
|  | Key stakeholders will be participants in the research (see above; WP5). However, in addition, we will also seek input from key stakeholders to steer the direction of our research and discuss the findings of the research and its implications. To achieve this we will hold three engagement events. We will hold one event before the research begins with parents of primary school aged children (key research participants) to explore our research plans and how we might adapt and develop them. We will hold two further events once findings are available, one with professionals (e.g. public health stakeholders from statutory and 3^rd^ sectors) and one with parents of primary school aged children. At these meetings we will seek views on our interpretation of findings and the implications of the findings for policy. After these meetings and finalisation of results, we will prepare written evidence briefings for national policymakers and retailers, as well as any research participants who request this during data collection. |
| **5)** | **Timetable of work:** |
|  | We estimate that this work will take 13 months to complete. Data collection and analysis will be conducted by a fieldworker at the University of Cambridge (WP1), a quantitative researcher at the University of Cambridge (WP2&3), and qualitative researchers at the University of Stirling (WP4&5). Data to be included in WP2&3 will be determined in WP1, meaning that WP2&3 will not start until WP1 results are available. To minimise time taken to complete the study, WP4&5 will start as early as possible.   |
| **6)** | **Outputs, anticipated contribution to the policy agenda and methods for disseminating findings:**  It is envisaged that the research will result in 4-5 research papers which we will publish in open access peer reviewed journals (such as International Journal of Behavioural Nutrition & Physical Activity, and Journal of Epidemiology & Community Health). In addition results will be disseminated at one national conference (e.g. Society for Social Medicine).  We will produce policy briefings for wide distribution to key stakeholders in the final month. These will be targeted to parents, the food retail sector, and public health policymakers and relevant civil society organisations. |
| **7)** | **Justification for support requested:** |
|  | We request the following support.  **1. Staff costs.** We will employ a fieldworker (new staff), quantitative researcher (new staff) and qualitative researchers (proportions of a number of existing staff) to complete this project. Using a fieldworker, rather than postdoctoral researcher, to collect data for WP1 reduces the costs. We also seek 10% of an administrator to contribute to focus group and interview organisation.  We also seek costs to support investigator time – 5%FTE for JA and 15% for MS who will directly supervise research staff; and 2.5%FTE each for MW and AJA who will contribute their expertise in evaluation of complex interventions and public health nutrition respectively.  **2. Equipment & software.** We request the costs of two digital audio recorders to enable data collection in WP1,4&5. We also request the costs of two desktop PCs – one for the quantitative researcher and one to contribute to qualitative data collection and analysis. The quantitative researcher will require a Stata licence, and the qualitative staff an NVivo licence to facilitate analysis.  **3. External services.** Data for WP2&3 will be supplied by Kantar Worldpanel at an estimated cost of £28,516.  **4. Fieldwork costs.** We requests funds for fieldwork travel to facilitate data collection for WP1&4. In addition, we will outsource transcription of interviews and focus groups in WP4&5 and request appropriate funds. Finally, we will outsource recruitment and hosting of focus groups in WP4 to an external market research company and seek funds for this, alongside venue hire and refreshments. We will reimburse all participants in WP4 with £20.  **5. Engagement.** We will build on PHRC’s existing relationship with the National Children’s Bureau to facilitate to the two engagement events with parents of primary school aged children. We request costs for the planned engagement event with professional stakeholders.  **6. Dissemination.** We request the costs of publishing five manuscripts in open access format to ensure our results are widely accessible. We will also present our results at two national conferences and request appropriate costs for one individual to attend each.  **7. Project management.** We will conduct the majority of project meetings by skype. However, we request funds for travel and catering of three in-person project meetings over the course of the project. |
| **8)** | References: |
|  | 1. Butland B, Jebb S, Kopelman P, McPherson K, Thomas S, Mardell J, et al. Foresight Tackling Obesities: future choices - project report. London: Government Office for Science; 2007.  2. Delmar-Morgan A. Sweets at supermarket tills are 'fuelling obesity crisis'. The Independent. 16 September 2013.  3. Children's Food Campaign. Checkouts checked out: How supermarkets and high street stores promote junk food to children and their parents 2012 [accessed 12 August 2014]. Available from: <http://www.sustainweb.org/publications/?id=212>.  4. Safefood. Safefood asks supermarkets to introduce healtheir checkouts Ireland13 February 2014 [accessed 28/05/2014]. Available from: <http://www.safefood.eu/News/2014/safefood-asks-supermarkets-to-introduce-healthier.aspx>.  5. Horsley JA, Absalom KAR, Akiens EM, Dunk RJ, Ferguson AM. The proportion of unhealthy foodstuffs children are exposed to at the checkout of convenience supermarkets. Public Health Nutrition. 2014;Epub ahead of print:1-6.  6. Thornton L, Cameron A, McNaughton S, Worsley A, Crawford D. The availability of snack food displays that may trigger impulse purchases in Melbourne supermarkets. BMC public health. 2012;12(1):194.  7. Miller C, Bodor JN, Rose D. Measuring the Food Environment: A Systematic Technique for Characterizing Food Stores Using Display Counts. Journal of Environmental and Public Health. 2012;2012:6.  8. Dixon H, Scully M, Parkinson K. Pester power: snackfoods displayed at supermarket checkouts in Melbourne, Australia. Health Promotion Journal of Australia. 2006;17(2):124-7.  9. Thornton L, Cameron A, McNaughton S, Waterlander W, Sodergren M, Svastisalee C, et al. Does the availability of snack foods in supermarkets vary internationally? International Journal of Behavioral Nutrition and Physical Activity. 2013;10(1):56.  10. Food Standards Agency. Nutrient Profiling: Food Standards Agency; 2010 [accessed 1 March 2010]. Available from: <http://www.food.gov.uk/healthiereating/advertisingtochildren/nutlab/>.  11. OfCom. Television advertising of food and drink products to children - final statement. London: Ofcom; 2007.  12. Wright J, Kamp E, White M, Adams J, Sowden S. Food at checkouts in non-food stores: a cross-sectional study of a large indoor shopping mall. Public Health Nutrition. 2015;FirstView:1-8.  13. Campbell S, James EL, Stacey FG, Bowman J, Chapman K, Kelly B. A mixed-method examination of food marketing directed towards children in Australian supermarkets. Health Promot Int. 2012;29(2):267-77.  14. van Kleef E, Otten K, van Trijp HC. Healthy snacks at the checkout counter: a lab and field study on the impact of shelf arrangement and assortment structure on consumer choices. BMC public health. 2012;12:1072.  15. Gilbert DC, Jackaria N. The efficacy of sales promotions in UK supermarkets: a consumer view. International Journal of Retail and Distribution Management. 2002;30(6):315-22.  16. Hawkes C. Sales promotions and food consumption. Nutrition Reviews. 2009;67(6):333-42.  17. Kontopantelis E, Doran T, Springate DA, Buchan I, Reeves D. Regression based quasi-experimental approach when randomisation is not an option: interrupted time series analysis. BMJ. 2015;350.  18. Zeger SL, Irizarry R, Peng RD. On time series analysis of public health and biomedical data. Annu Rev Public Health. 2006;27:57-79.  19. Gale N, Heath G, Cameron E, Rashid S, Redwood S. Using the framework method for the analysis of qualitative data in multi-disciplinary health research. BMC Medical Research Methodology. 2013;13(1):117. |

| C | CVs OF PI AND CIs (maximum 1 page) | | | | | | | |
| --- | --- | --- | --- | --- | --- | --- | --- | --- |
| CURRICULUM VITAE | | | | | | | | |
| *Name:* | | Martin White | | | | | | |
| *Title:* | | Prof | | | | | | |
| *Position:* | | Programme Leader, Centre for Diet & Activity Research, MRC Epidemiology Unit, University of Cambridge | | | | | | |
| Qualifications: | | | | | | | | |
| *Qualification:* | | | | *Awarding body:* | | | *Date:* | |
| MD (Public Health) | | | | Birmingham University | | | 2010 | |
| FFPH (Fellowship of the Faculty of Public Health) | | | | Faculty of Public Health | | | 1997 | |
| MSc (Public Health) | | | | Newcastle University | | | 1989 | |
| MB ChB (Medicine) | | | | Birmingham University | | | 1983 | |
| Present and previous positions: | | | | | | | | |
| *Position:* | | | *Organisation:* | | | | *Dates:* | |
| *Present:* Director of Research and Programme Leader | | | Centre for Diet & Activity Research, MRC Epidemiology Unit, University of Cambridge | | | | 2014 – present | |
| Director | | | NIHR Public Health Research Programme | | | | 2014-19 | |
| Honorary Consultant in Public Health | | | Public Health England | | | | 2103 – present | |
| *Previous:* Director | | | Fuse, UKCRC Centre for Translational Research in Public Health | | | | 2008-14 | |
| Professor of Public Health | | | Institute of Health & Society, Newcastle University | | | | 2005-14 | |
| Current & recent research grants (selected): | | | | | | | | |
| *Title:* | | | | *Role:* | *Funder:* | *Amount:* | | *Dates:* |
| Evaluation of the impact of a levy on added-sugar soft drinks on sales and purchasing behaviour within *Jamie’s Italian* restaurants | | | | Co-I | NIHR Public Health Research Programme | £47,480 | | 2015 (6 mths) |
| NHS Diabetes Prevention Programme: process evaluation of the demonstrator phase & consensus building for a common evaluation framework | | | | Co-I | NIHR School of Public Health Research | £250,000 | | 2015 (18 mths) |
| Transforming the ‘foodscape’: development and feasibility testing of interventions to promote healthier take-away, pub or restaurant food | | | | Co-I | NIHR School of Public Health Research | £446,646 | | 2013 (30 mths) |
| Phase 2 research to support the evaluation and implementation of adult cooking skills interventions in the UK: pilot RCT with process and economic evaluation components | | | | PI | Public Health Research Consortium | £355,849 | | 2013 (20 mths) |
| Recent relevant publications (selected): | | | | | | | | |
| [Adams J](http://www.ncbi.nlm.nih.gov/pubmed/?term=Adams%20J%5BAuthor%5D&cauthor=true&cauthor_uid=26242297) et al. Prevalence and socio-demographic correlates of cooking skills in UK adults: cross-sectional analysis of data from the UK National Diet and Nutrition Survey. [*Int J Behav Nutr Phys Act.*](http://www.ncbi.nlm.nih.gov/pubmed/26242297?dopt=Abstract&holding=f1000,f1000m,isrctn) 2015;12(1): 99. | | | | | | | | |
| Wright J et al (2015) Food at checkouts in non-food stores: a cross-sectional study of a large indoor shopping mall *Public Health Nutrition* doi:10.1017/S1368980015000178 | | | | | | | | |
| McGill R et al. Are interventions to promote healthy eating equally effective for all? Systematic review of socio-economic inequalities in impact. *BMC Public Health,* 2015; **15**: 457 | | | | | | | | |
| Lara J et al., Association of behaviour change techniques with effectiveness of dietary interventions among adults of retirement age: a systematic review and meta-analysis of randomised controlled trials. *BMC Medicine* 2014, 12:177. | | | | | | | | |
| Howard S et al., Nutritional content of supermarket ready meals and recipes by television chefs in the United Kingdom: cross sectional study. *BMJ* 2012; 45: e7607. | | | | | | | | |

| CURRICULUM VITAE | | | | | | | |
| --- | --- | --- | --- | --- | --- | --- | --- |
| *Name:* | Jean Adams | | | | | | |
| *Title:* | Dr | | | | | | |
| *Position:* | Programme Leader, Centre for Diet & Activity Research, MRC Epidemiology Unit, University of Cambridge | | | | | | |
| Qualifications: | | | | | | | |
| *Qualification:* | | | *Awarding body:* | | | *Date:* | |
| PGCert, Science Communication | | | University of the West of England | | | 2012 | |
| MSc, Health Psychology | | | City University, London | | | 2007 | |
| PhD, Epidemiology & Public Health | | | Newcastle University | | | 2004 | |
| MBBS, Medicine | | | Newcastle University | | | 2001 | |
| BMedSci (Hons), Health Psychology and Psychiatry | | | Newcastle University | | | 1998 | |
| Present and previous positions: | | | | | | | |
| *Position:* | | *Organisation:* | | | | *Dates:* | |
| Senior Research Fellow | | University of Cambridge | | | | 2014-present | |
| Senior Lecturer in Public Health | | Newcastle University | | | | 2012-14 | |
| NIHR Career Development Fellow | | Newcastle University, then University of Cambridge | | | | 2012-15 | |
| Lecturer in Public Health | | Newcastle University | | | | 2008-12 | |
| MRC Special Training Fellow | | Newcastle University | | | | 2005-09 | |
| Current & recent research grants (selected): | | | | | | | |
| *Title:* | | | *Role:* | *Funder:* | *Amount:* | | *Dates:* |
| Evaluation of the impact of a levy on added-sugar soft drinks on sales and purchasing behaviour within *Jamie’s Italian* restaurants | | | Co-I | NIHR Public Health Research Programme | £47,480 | | 2015 (6 mths) |
| Determinants and health outcomes of home food preparation | | | PI | NIHR Doctoral Research Fellowship (Dr S Mills) | £278,679 | | 2014 (36 mths) |
| Transforming the ‘foodscape’: development and feasibility testing of interventions to promote healthier take-away food | | | Co-I | NIHR School of Public Health Research | £446,646 | | 2013 (30 mths) |
| Phase 2 research to support the evaluation and implementation of adult cooking skills interventions in the UK: pilot RCT with process and economic evaluation | | | PI | Public Health Research Consortium | £355,849 | | 2013 (20 mths) |
| Recent relevant publications (selected): | | | | | | | |
| [Adams J](http://www.ncbi.nlm.nih.gov/pubmed/?term=Adams%20J%5BAuthor%5D&cauthor=true&cauthor_uid=26242297) et al. Prevalence and socio-demographic correlates of cooking skills in UK adults: cross-sectional analysis of data from the UK National Diet and Nutrition Survey. [*Int J Behav Nutr Phys Act.*](http://www.ncbi.nlm.nih.gov/pubmed/26242297?dopt=Abstract&holding=f1000,f1000m,isrctn) 2015;12(1): 99. | | | | | | | |
| Wright J et al (2015) Food at checkouts in non-food stores: a cross-sectional study of a large indoor shopping mall *Public Health Nutrition* doi:10.1017/S1368980015000178 | | | | | | | |
| Adams J, Goffe L, Brown T, Lake AA, Summerbell C, White M, Wrieden W, Adamson AJ (2015) Frequency and socio-demographic correlates of eating meals out and take-away meals at home: cross-sectional analysis of the UK National Diet and Nutrition Survey, waves 1-4. International Journal of Behavioural Nutrition & Physical Activity 12:51 doi:10.1186/s12966-015-0210-8 | | | | | | | |
| Remnant J & Adams J (2015) The nutritional content and cost of supermarket ready-meals: cross-sectional analysis. Appetite 92:36-42 doi:10.1016/j.appet.2015.04.069 | | | | | | | |
| Howard S et al., Nutritional content of supermarket ready meals and recipes by television chefs in the United Kingdom: cross sectional study. *BMJ* 2012; 45: e7607. | | | | | | | |

| CURRICULUM VITAE | | | | | | | |
| --- | --- | --- | --- | --- | --- | --- | --- |
| *Name:* | Martine Stead | | | | | | |
| *Title:* | Ms | | | | | | |
| *Position:* | Deputy Director, Institute for Social Marketing (ISM), University of Stirling | | | | | | |
| Qualifications: | | | | | | | |
| *Qualification:* | | | *Awarding body:* | | | *Date:* | |
| BA (Hons) English | | | UCL, University of London | | | 1987 | |
| Present and previous positions: | | | | | | | |
| *Position:* | | *Organisation:* | | | | *Dates:* | |
| Deputy Director, Institute for Social Marketing (ISM) | | University of Stirling | | | | 2005-present | |
| Senior Researcher, Centre for Social Marketing | | University of Strathclyde | | | | 1997-2005 | |
| Research Officer, Centre for Social Marketing | | University of Strathclyde | | | | 1992-7 | |
| Health Promotion Resource Development Officer | | Ealing Health Authority | | | | 1990-2 | |
| Current & recent research grants (selected): | | | | | | | |
| *Title:* | | | *Role:* | *Funder:* | *Amount:* | | *Dates:* |
| Mass Media for Public Health Messages | | | Co-I | NIHR | £212,037 | | 2015 (18 mths) |
| Evaluation of free school meals in Scotland. | | | Co-I | NHS Health Scotland | £70,000 | | 2015 (12 mths) |
| Tobacco marketing in the EU | | | Co-I | EU DG Sante/CHAFEA | £153,807 | | 2014 (12 mths) |
| Family history and cancer risk | | | Co-I | Dundee Cancer Centre | £30,000 | | 2013 (15 mths) |
| Process Evaluation of Alcohol Brief Interventions in wider settings | | | PI | NHS Health Scotland | £79,996 | | 2012 (11 mths) |
| ALICE RAP - Addictions and lifestyles in contemporary Europe - Reframing Addictions Project | | | Co-I | European Commission | £103,714 | | 2011 (60 mths) |
| BeWEL – The impact of a bodyweight and physical activity intervention on adults at risk of developing colorectal adenoma | | | Co-I | MRC (though University of Dundee) | £42,764 | | 2010 (36 mths) |
| Recent relevant publications (selected): | | | | | | | |
| Eadie D, **Stead M**, MacKintosh AM, Macdonald L, Purves R, Pearce J, Tisch C, van der Sluijis W, Amos A, MacGregor A and Haw S (2015). E-cigarette marketing in UK stores: An observational audit and retailers’ views. BMJ Open, 5(9):e008547, | | | | | | | |
| Haw S, Amos A, Eadie D, Frank J, Macdonald L, MacKintosh AM, MacGregor A, Miller M, Pearce J, Sharp C, **Stead M**, Tisch C and van der Sluijs W (2014). Determining the impact of smoking point of sale legislation among youth (Display) study: A protocol for an evaluation of public health policy. BMC Public Health, 14(1): 251. http://www.biomedcentral.com/content/pdf/1471-2458-14-251.pdf | | | | | | | |
| Anderson AS, Craigie AM, Caswell S, Treweek S, **Stead M**, Macleod M, Daly F, Belch J, Rodger J, Kirk A, Ludbrook A, Rauchhaus P, Norwood P, Thompson J, Wardle J and Steele RJ (2014). The impact of a bodyweight and physical activity intervention (BeWEL) initiated through a national colorectal cancer screening programme: Randomised controlled trial. British Medical Journal, 348: g1823, doi: 10.1136/bmj.g1823. http://www.bmj.com/content/348/bmj.g1823 | | | | | | | |
| Anderson AS, Freeman J, **Stead M**, Wrieden WL, Barton KL. Consumer views on portion size guidance to assist adult dietary choices. *Journal of Human Nutrition and Dietetics* 2008; 21(4): 375. | | | | | | | |
| **Stead M**, Caraher M, Wrieden W, Longbottom P, Valentine K and Anderson A.  Confident, fearful and hopeless cooks: Findings from the development of a food-skills initiative.  *British Food Journal* 2004; 106(4): 274-287. | | | | | | | |

| CURRICULUM VITAE | | | | | | | |
| --- | --- | --- | --- | --- | --- | --- | --- |
| *Name:* | Ashley Adamson | | | | | | |
| *Title:* | Prof | | | | | | |
| *Position:* | Professor of Public Health Nutrition, Institute of Health & Society, Newcastle University | | | | | | |
| Qualifications: | | | | | | | |
| *Qualification:* | | *Awarding body:* | | | | *Date:* | |
| PhD, Public Health Nutrition | | Newcastle University | | | | 1993 | |
| BSc Nutrition & Registration in Dietetics | | Queen Margaret College, Edinburgh | | | | 1987 | |
| Fellow of Faculty of Public Health by distinction | | Faculty of Public Health | | | | 2011 | |
| Present and previous positions: | | | | | | | |
| *Position:* | | | | *Organisation:* | | *Dates:* | |
| NIHR Research Professor and Professor of Public Health Nutrition - Director of Fuse UKCRC Centre for Translation Research in Public Health (from Oct 2014) | | | | Newcastle University | | 2013-present | |
| Professor of Public Health Nutrition - Programme director for Public Health and Applied Health Interventions & deputy director of the Human Nutrition Research Centre | | | | Newcastle University | | 2009-13 | |
| SL Public Health Nutrition, Human Nutrition Research Centre and Institute of Health and Society | | | | Newcastle University | | 2005-09 | |
| Current & recent research grants (selected): | | | | | | | |
| *Title:* | | *Role:* | *Funder:* | | *Amount:* | | *Dates:* |
| How can we help parents recognise unhealthy body weight in their children? | | PI | MRC-NPRI | | £471 298 | | 2012 (34 mths) |
| Transforming the ‘foodscape’: development and feasibility testing of interventions to promote healthier take-away, pub or restaurant food | | PI | NIHR-SPHR (total £2.5M 2012-2017) | | £446,646 | | 2013 (30 mths) |
| NIHR Research Professor personal award | | PI | NIHR | | £1.2M | | 2013 60 mths) |
| Fuse - The Centre for Translational Research in Public Health | | PI | UKCRC | | £3.9M | | 2013 (60 mths) |
| Phase 2 research to support the evaluation and implementation of adult cooking skills interventions in the UK: pilot RCT with process and economic evaluation components | | Co-I | PHRC | | £367,914 | | 2013 (20 mths) |
| Recent relevant publications (selected): | | | | | | | |
| Adams J, Goffe L, Brown T, Lake AA, Summerbell C, White M, Wrieden W, Adamson AJ: Frequency and socio-demographic correlates of eating meals out and take-away meals at home: cross-sectional analysis of the UK national diet and nutrition survey, waves 1–4 (2008–12), *International Journal of Behavioral Nutrition and Physical Activity*, 2015, 12:51 | | | | | | | |
| Hillier-Brown FC, Moore HJ, Lake AA, Adamson AJ, White M, Adams J, Araujo-Soares V, Abraham C, Summerbell CD: The effectiveness of interventions targeting specific out-of-home food outlets: protocol for a systematic review. *Systematic Reviews,* 2014, 3(17) | | | | | | | |
| Howel D, Stamp E, Chadwick TJ, Adamson AJ, White M: Are social inequalities widening in generalised and abdominal obesity and overweight among English Adults? *PLoS One,* 2013, 8:e79027 | | | | | | | |
| Craigie AM, Lake AA, Kelly SA, Adamson AJ, Mathers JC: Tracking of obesity-related behaviours from childhood to adulthood: A systematic review *Maturitas* 2011, 70:266-284 | | | | | | | |
| Jones AR, Parkinson KN, Drewett RF, Hyland RM, Pearce MS, Adamson AJ and the Gateshead Millennium Study core team. Parental perceptions of weight status in children: the Gateshead Millennium Study'. *International Journal of Obesity* 2011; 35(7), pp. 953-962. | | | | | | | |

| **D** | **COSTS** |
| --- | --- |
|  | |
| **SEE ATTACHED PHRC BUDGET FORM** | |
